# Chlorinated bis-4-hydroxycoumarins suppress flavivirus replication by inhibiting dengue virus type 2 translation and replication

**DOI:** 10.1038/s41598-026-35654-8

**Published:** 2026-01-16

**Authors:** Naphat Loeanurit, Thi-Hong-Truc Phan, Kowit Hengphasatporn, Thanchanok Chanachanvong, Borwornlak Toopradab, Phornphimon Maitarad, Prangwalai Chanchaem, Vorthon Sawaswong, Sunchai Payungporn, Ajirawadee Suwanchan, Auwal Rabiu Auwal, Kittikhun Wangkanont, Thanyada Rungrotmongkol, Yasuteru Shigeta, Tanatorn Khotavivattana, Warinthorn Chavasiri, Siwaporn Boonyasuppayakorn

**Affiliations:** 1 Center of Excellence in Applied Medical Virology, Department of Microbiology, Faculty of Medicine, Chulalongkorn University, Bangkok, 10330 Thailand; 2 Center of Excellence in Natural Products Chemistry, Department of Chemistry, Faculty of Science, Chulalongkorn University, Bangkok, 10330 Thailand; 3 Center for Computational Sciences, University of Tsukuba, 1-1-1 Tennodai, Tsukuba, Ibaraki 305-8577 Japan; 4 Program in Bioinformatics and Computational Biology, Graduate School, Chulalongkorn University, Bangkok, 10330 Thailand; 5 Research Center of Nano Science and Technology, Department of Chemistry, College of Science, Shanghai University, Shanghai, 200444 People’s Republic of China; 6 Center of Excellence in Systems Microbiology, Department of Biochemistry, Faculty of Medicine, Chulalongkorn University, Bangkok, 10330 Thailand; 7 Center of Excellence for Molecular Biology and Genomics of Shrimp, Department of Biochemistry, Faculty of Science, Chulalongkorn University, Bangkok, 10330 Thailand; 8 Center of Excellence in Molecular Crop, Department of Biochemistry, Faculty of Science, Chulalongkorn University, Bangkok, 10330 Thailand; 9 Center of Excellence in Biocatalyst and Sustainable Biotechnology, Department of Biochemistry, Faculty of Science, Chulalongkorn University, Bangkok, 10330 Thailand

**Keywords:** Biochemistry, Chemical biology, Drug discovery, Microbiology

## Abstract

Dengue virus (DENV) remains a major global health threat, and no clinically approved antiviral therapy is currently available. Halogenated biscoumarins have been reported as versatile antimicrobial and antiviral agents. Here, we screened eleven halogenated biscoumarin derivatives against DENV and identified compounds 3 and 4—bearing chlorine substitutions at the 3- or 4-position of the phenyl moiety—as the most potent inhibitors of DENV2, with EC_50_ values of 3.62–9.72 µM and 4.62–10.88 µM, respectively, and selectivity indices (SI) up to 20.97. Both compounds also inhibited all four DENV serotypes and Zika virus (ZIKV) with comparable efficacy. Mechanistic analyses demonstrated that compounds 3 and 4 significantly suppressed viral translation and RNA replication in infectious and replicon models. Long-term passaging generated mutations in NS4B, although these substitutions did not confer resistance, was involved in self-dimerization to curvature formation of ER-derived spherules, which may reflect adaptive changes linked to host interaction. In vitro enzymatic assays further indicated that the viral NS5 methyltransferase is a potential target, with IC_50_ values of 4.60 ± 0.83 µM and 3.17 ± 0.25 µM for compounds 3 and 4, respectively, and both compounds inhibited NS3 protease activity by > 89% at 50 µM. Collectively, these results identify chlorinated biscoumarins as promising antiviral scaffolds that impair flaviviral translation and replication, supporting their further optimization and in vivo evaluation.

**Supplementary Information:**

The online version contains supplementary material available at 10.1038/s41598-026-35654-8.

## Introduction

Dengue virus, a single-stranded, positive-sense RNA flavivirus, undergoes a complex life cycle within mosquito vectors and human hosts. The genome encodes three structural and seven nonstructural proteins for virion formation, replication, RNA synthesis, and immune modulation^1^. The virus enters the cells via receptor-mediated endocytosis and uncoats during pH-dependent fusion. The viral genome directs host translational machinery to translate a polyprotein, which is subsequently cleaved by host and viral proteases^1^. Replication occurs within the ER-derived pockets in the cytoplasm using an NS3-NS5 replicase complex. The cryoelectron microscopy (cryo-EM) revealed that a V-shaped RNA element of 5′-stem-loop structure (SLA) bridged the NS5 methyltransferase and RNA-dependent RNA polymerase domains (NS5-SLA complex) to initiate replication and RNA capping^2^. Later, the NS3 helicase domain is incorporated into the complex (NS5-NS3-SLA complex), thus rearranging the NS5 domains and displacing the SLA into the elongation complex (EC). Subsequently, the genome is assembled into the virion and undergoes maturation (including prM protein cleavage to M protein) while traveling through the Golgi complex before the release of infectious virions^1^. Dengue pathogenesis is the interplay between the virus and the immune system, triggering robust but incompetent immunological enhancement that leads to severe hemorrhagic fever^3^. Current dengue vaccines, such as Qdenga and Dengvaxia, are live attenuated vaccines that offer partial protection against all four dengue virus serotypes by reducing the chance of infection and the severity of the disease. Antiviral agents are still in need for treatment of acute dengue to prevent dysfunctional immune responses and increasing viral loads. Elucidating the molecular mechanisms of dengue virus is vital for the development of diagnostics, therapeutics, and vaccines to effectively combat this global public health concern.

Natural products represent a vast and historically significant source of antimicrobial compounds. Coumarins, characterized by their fused benzene and α-pyrone ring structure, are abundant secondary metabolites found in various plant families. These compounds demonstrate a wide array of biological activities, including antiviral properties, attributable to their versatile structures^4^. Research indicates that coumarins act as broad-spectrum antivirals against HIV, herpes simplex, enteroviruses, hepatitis, dengue (DENV), Zika (ZIKV), chikungunya (CHIKV), and influenza^4–9^. Coumarin-based inhibitors such as ramosin, myresellinol, myresellin, and thiazolidinone coumarins, target viral proteases and polymerases and interfere with viral replication (review by^10^. Recently, another set of coumarin derivatives called biscoumarins has gained interest in their potential therapeutic effects, according to their noncovalent interactions with various active sites^11^. Moreover, their low toxicity and stability in aqueous solutions offer advantages for pharmacokinetic studies^4^. In this study, we screened biscoumarin analogs previously characterized^12^ against DENV and ZIKV, and characterized the potential compounds for further investigation. The selected compounds inhibited all serotypes of DENV and ZIKV with good efficacies. Moreover, a pipeline of virtual and cell-based assays characterized the viral methyltransferase as one of the potential targets.

## Results

### Biscoumarin derivatives exhibit antiviral activities against DENV2 and ZIKV activity

Twelve biscoumarin analogs (Table 1, Supplementary Tables S1-S2) were synthesized and identified as previously described^12^. We first performed a primary antiviral screen against DENV2 and ZIKV at an MOI of 0.1. DENV serotype 2 was used as the representative strain in subsequent analyses because secondary DENV2 infection is consistently associated with severe clinical outcomes^13–15^. For the primary assay, Vero cells infected with DENV2 or ZIKV were treated with each compound at 10 µM and incubated for 3 or 2 days, respectively, before supernatants were collected for plaque titration. DMSO served as the no-inhibition control. Cytotoxicity was evaluated in parallel by treating uninfected Vero cells with each compound (10 µM) and assessing cell viability by MTS assay. Compounds 3, 4, and 8 demonstrated the strongest antiviral activities, reducing infectious DENV2 and ZIKV production by ≥ 90% while maintaining > 90% cell viability. Their antiviral efficacy markedly exceeded that of the parent compound 1, which possesses an unsubstituted phenyl ring and inhibited only 48–50% of viral replication. This structure–activity relationship suggests that halogen substitution enhances antiviral potency. All active compounds contained a chlorine atom at the 3-, 4-, or both positions of the phenyl substituent. In contrast, 2-chlorinated (compound 2) and 2,4-dichlorinated (compound 7) derivatives showed limited activity against ZIKV. Replacing the 4-Cl group with fluorine or bromine (compounds 5 and 6) similarly reduced antiviral efficacy, particularly against ZIKV. Likewise, modifying the 3-Cl position to 3-NO₂ or 3-Br (compounds 9 and 10–12) attenuated inhibition of both DENV2 and ZIKV. Compounds 3, 4, and 8 were advanced from the primary screen because they achieved ≥ 90% inhibition of both DENV2 and ZIKV at 10 µM while maintaining > 90% cell viability.

Together, these findings support the conclusion that chlorine substitution at the 3- and/or 4-position is critical for antiviral activity. Based on the primary screen, compounds 3, 4, and 8 were selected for expanded cytotoxicity profiling across multiple human and non-human cell lines (Table 3). This secondary evaluation revealed that compound 8 exhibited substantially higher cytotoxicity and therefore a markedly lower selectivity index compared with compounds 3 and 4. Because only compounds 3 and 4 met the criteria for favorable antiviral potency and acceptable cytotoxicity, they were advanced for mechanistic studies, including molecular docking and MD simulations. Compound 8 was excluded from subsequent mechanistic studies due to its substantially higher cytotoxicity in human-derived cell lines, resulting in the lowest selectivity index among active compounds.

**Table 1:** Primary screening of twelve biscoumarin derivatives against DENV2 and ZIKV. Vero cells were infected with DENV2 or ZIKV at MOI of 0.1 and the test compounds (at 10 µM) were added during and after infection. DMSO was used as no-inhibition control. The culture supernatants were collected to quantify viral titers. The results were calculated as percent Inhibition relative to DMSO control. Vero cell viability was quantified in parallel by adding 10 µM of each compound to Vero cells. Cell viability was measured using MTS assay and calculated as percent of cell viability, with DMSO control defined as 100% cell viability. Data represents the mean ± standard deviation of triplicate results.

No.		Cell viability (%)	Viral inhibition (%)	
	Substitution (**R**)	Vero	DENV2	ZIKV
**1**	-	101.01 ± 1.15	48.00 ± 24.26	50.00 ± 25.00
**2**	2-Cl	97.51 ± 2.03	95.07 ± 0.82	69.23 ± 15.38
**3**	3-Cl	97.00 ± 1.08	99.56 ± 0.14	98.85 ± 0.08
**4**	4-Cl	95.44 ± 1.31	99.27 ± 0.05	94.23 ± 2.69
**5**	4-F	95.78 ± 0.42	−13.33 ± 16.44	−15.38 ± 30.77
**6**	4-Br	97.00 ± 0.27	97.60 ± 0.57	73.08 ± 3.85
**7**	2,4-diCl	99.62 ± 0.51	96.93 ± 0.19	42.31 ± 3.85
**8**	3,4-diCl	94.89 ± 1.48	99.99 ± 0.02	99.88 ± 0.04
**9**	3-NO_2_	95.38 ± 1.76	75.00 ± 6.62	7.69 ± 30.77
**10**	3-Br, 4-OH	97.09 ± 2.23	17.33 ± 6.80	−7.69 ± 15.38
**11**	3-Br, 4-OCH_3_	100.04 ± 5.61	−24.00 ± 18.18	0.00 ± 7.69
**12**	3,5-diBr, 4-OH	98.35 ± 0.98	18.67 ± 14.73	−23.08 ± 15.38

### QSAR modeling reveals structural determinants that correlate with antiviral activity

Quantitative structure–activity relationship (QSAR) modeling establishes a quantitative relationship between molecular descriptors and biological activity. This relationship enables the prediction of important compound properties, thereby accelerating the drug discovery process. In the initial stage of QSAR modeling, the classical QSAR with genetic function approximation-multiple linear regression (GFA-MLR) model of biscoumarins against DENV2 is shown in Eq. 1. The square correlation coefficient (*R*^2^) value was 0.464, and the cross-validation (*R*^2^_CV_) with a value of -0.396.

1 $$\begin{aligned} {\mathrm{log}}\left[ {\% {\mathrm{inhibition}}} \right]{\text{ }} & = ~ - {\text{ }}0.{\text{339 }}*{\text{ Total dipole}} \\ &\quad + {\text{ }}0.{\text{433 }}*{\text{ Rotatable bonds}} + {\text{ 1}}.{\mathrm{484}} \\ \end{aligned}$$

For ZIKV, the GFA-ML model is shown in Eq. 2. The *R*^2^ value was 0.840, and the *R*^2^_CV_ was 0.592.

2 $$\begin{aligned} {\mathrm{log}}\left[ {\% {\mathrm{inhibition}}} \right]{\text{ }} & = ~ - {\text{ 1}}.0{\text{46 }}*{\text{ Rotatable bonds}} \\ & \quad + {\text{ }}0.{\text{578 }}*{\text{ E}} - {\text{state keys }}\left( {{\mathrm{sums}}} \right):{\text{ S}}\_{\mathrm{aasC}} \\ & \quad + {\text{ 1}}.{\mathrm{1}}0{\mathrm{7}} \\ \end{aligned}$$

These classical QSAR results were not effective in describing the relationship between molecular structures and biological efficiency. Therefore, we explored alternative approaches by utilizing machine learning-based regression techniques. All molecular descriptors were selected as features of importance to elucidate the QSAR-ML model using the Gini Importance (GI), as shown in supplementary Tables S3-S5. The common descriptors are based on the GI results of DENV2 and ZIKV targets shown in Table 2. For DENV2, the GI results showed that the dipole y had a low value of 0.100, and the molecular area had a value of 0.122. For ZIKV, the GI results indicated that the molecular area had a value of 0.150, and dipole y had a value of 0.264. The correlation coefficient between dipole y and molecular area was 0.277. Therefore, for both DENV2 and ZIKV, verifying the GI values and correlation coefficient values of selected descriptors indicated appropriate GI values and the absence of high correlation coefficients.

**Table 2:** Main descriptors and their definition for biscoumarin derivatives against each virus.

Descriptor	Definition	GI value	
		DENV2	ZIKV
Dipole y	Y-axis component of the molecular dipole moment	0.100	0.264
Molecular area	van der Waals (vdW) surface area of the molecule	0.122	0.150

**Fig. 1:**
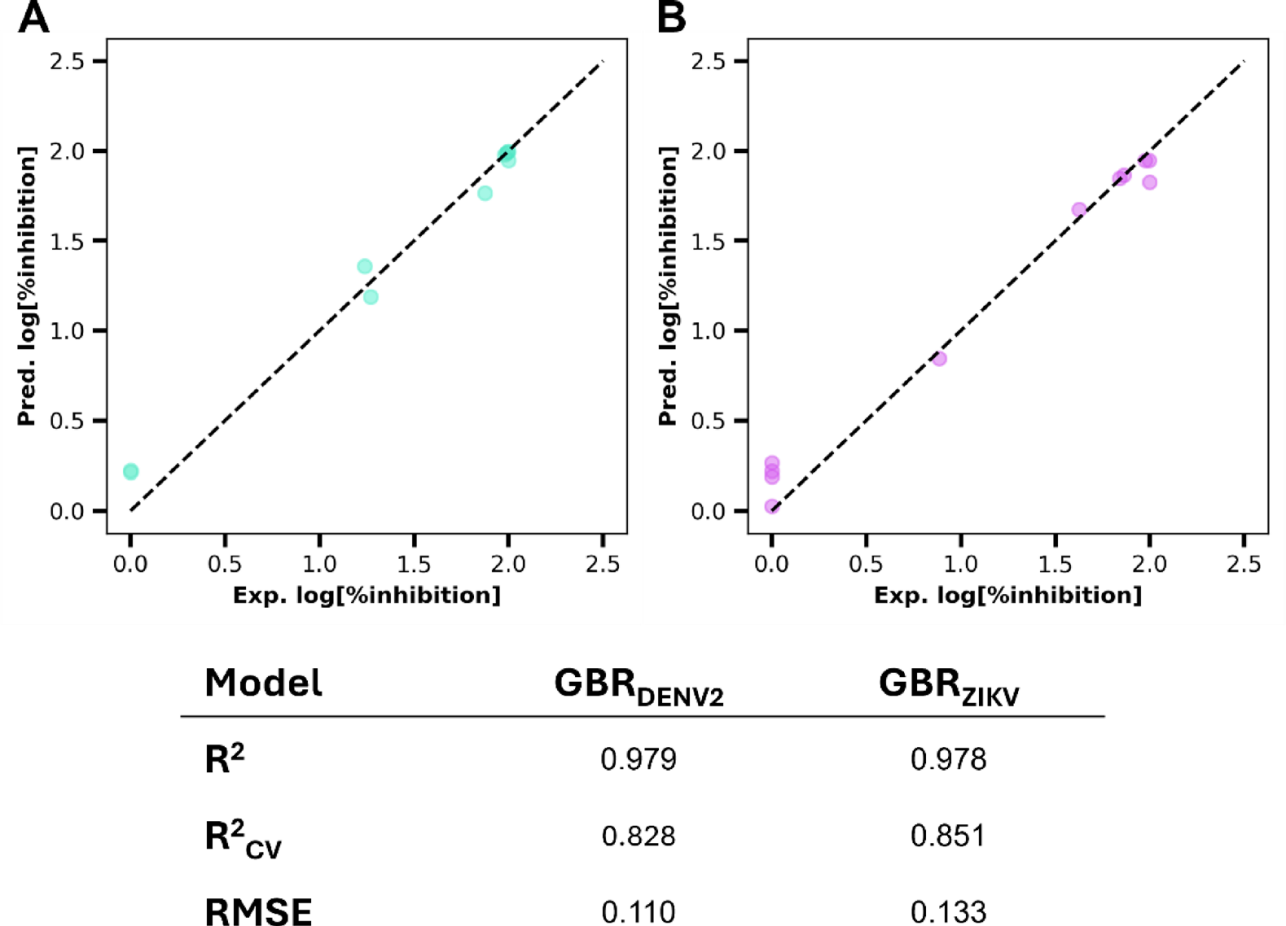
Correlation between experimental and predicted log[%inhibition] values of biscoumarin derivatives against (**A**) DENV2 and (**B**) ZIKV, as determined by the GBR models.

The Random Forest (RF) and Gradient Boosting Regression (GBR) algorithms were employed to construct QSAR-ML models for DENV2 and ZIKV. Model performance was assessed using the coefficient of determination (R^2^), leave-one-out cross-validation (*R*^2^_cv_), and Root Mean Square Error (RMSE). The GBR model for DENV2 achieved *R*^2^, *R*^2^_cv_, and RMSE values of 0.979, 0.828, and 0.110, respectively. Similarly, the GBR model for ZIKV yielded *R*^2^, *R*^2^_cv_, and RMSE values of 0.978, 0.851, and 0.133, respectively. These results, presented in Fig. 1, indicate that the GBR models exhibit strong predictive performance for both viruses and are likely superior to the RF models (Supplementary Table S6). Predicted values for all biscoumarin compounds are listed in Supplementary Table S7.

In summary, classical QSAR modeling was insufficient to capture the relationship between molecular features and antiviral activity; however, machine learning–based approaches markedly improved predictive accuracy. Gradient boosting regression models achieved strong correlations with experimental data for both DENV2 and ZIKV, highlighting their advantage over traditional methods. Descriptor analysis identified the y-component of the dipole moment and molecular surface area as key contributors to antiviral potency across both viruses. These results underscore the utility of ML-enhanced QSAR modeling for guiding future structural optimization of biscoumarin derivatives.

### Molecular Docking and MD simulation identify NS5 methyltransferase as the primary target of chlorinated biscoumarins

**Fig. 2:**
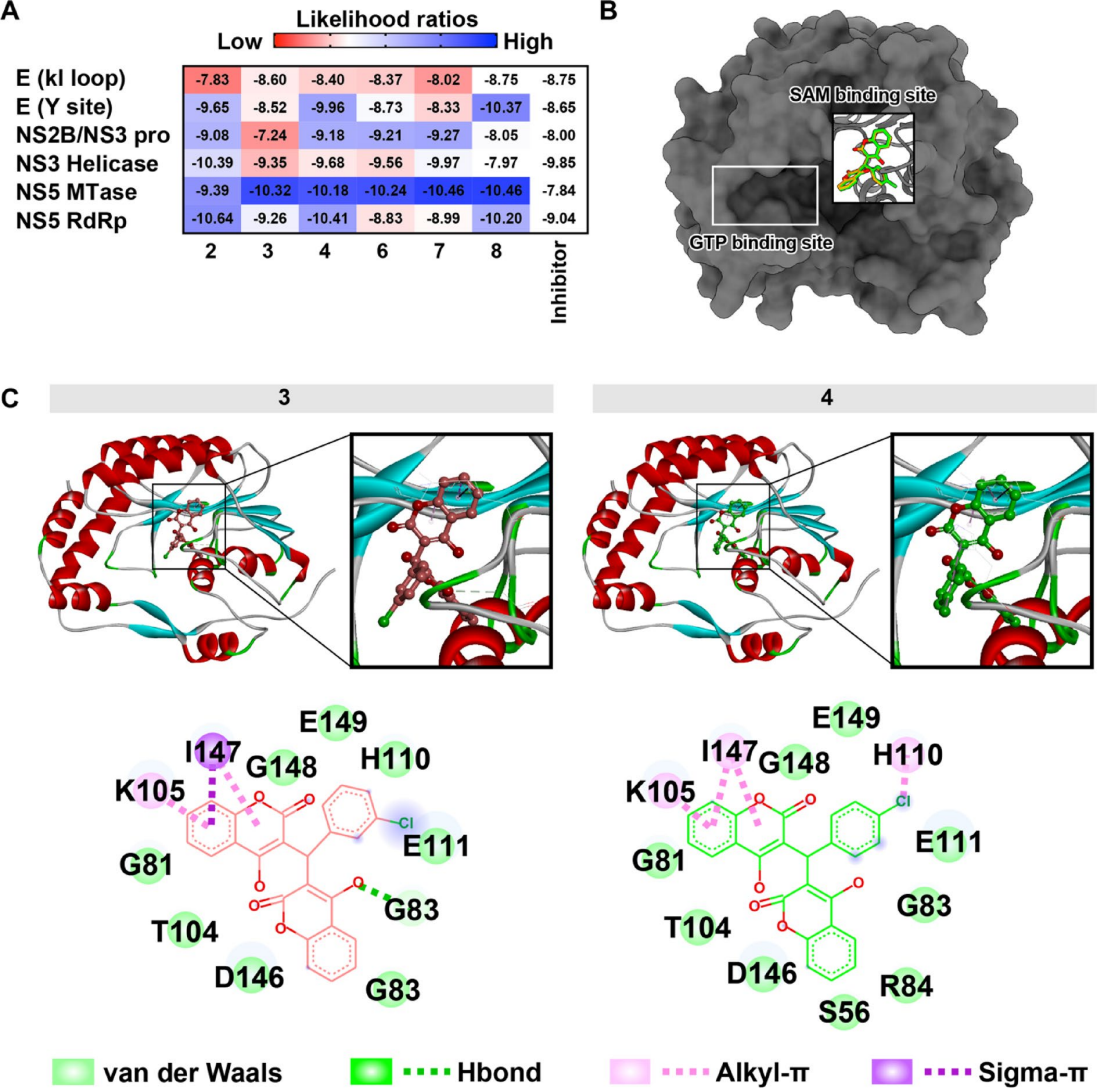
The molecular docking results of biscoumarin analogs against the target viral protein were compared to the native inhibitor of each protein. (**A**) In the grid plot, colors indicate the likelihood of the compounds targeting the viral protein, ranging from low (red) to high (blue), with white representing reference data from the known inhibitor. The numerical values represent the AutoDock Vina docking scores (kcal/mol). (**B**) The binding sites of biscoumarins on NS5 MTase are highlighted, including the molecular results for **3** and **4**. (**C**) The 2D and 3D interaction views depict these biscoumarin analogs engaging with the SAM binding pocket of NS5 MTase.

In addition, the halogenated compounds were screened using in silico pan-docking to predict the possibility of the viral target. The proteins used in docking were the main proteins involved in the viral replication cycle, including envelope (E) protein (1OKE), the allosteric site of NS2B/NS3 protease (NS2B/NS3 pro) (2FOM), ATP binding site of NS3 helicase (NS3 helicase) (2BHR), the SAM binding site of NS5 methyltransferases (NS5 MTase) (3EVG), and NS5 RNA-dependent RNA polymerase (NS5 RdRp) of DENV2 (6IZX). By comparing the native inhibitor for each viral target, the results showed that the possible target could be the DENV2 NS5 MTase (Fig. 2). Compound **3** showed the strongest binding energy to NS5 MTase, similar to the results of compound **3** from the primary screening (Fig. 2A). Therefore, the viral MTase might be a target of compound **3**. Moreover, the interactions between compound **3** and MTase showed that compound **3** exhibited the lowest binding energy at − 10.32 kcal/mol, lower than the binding energy of sinefugin (− 7.84 kcal/mol), indicating a more favorable interaction with the target compared to the reference compound (Fig. 2A). A 3D structural analysis revealed that both compounds **3** and **4** bind to the target protein at the SAM binding site, similar to sinefungin (Fig. 2B), forming specific contacts with defined residues rather than a general set of amino acids. Compound 3 engages in hydrogen bonds with E111, G83, T104, and D146, while also establishing alkyl–π interactions with I147 and G148 and a σ–π interaction with K105. Compound 4 exhibits a similar hydrogen-bonding pattern with E111, G83, T104, and D146, but differs in its π-related contacts, forming additional interactions with H110, R84, and S56. These residue-resolved interactions contribute to the stability of the complex between the compound and the target protein (Fig. 2C). When compared to compound **4**, which exhibited a similar binding energy, the distinct chemical bonds suggest a different binding specificity (Fig. 2C). In addition to NS5 MTase, potential antiviral mechanisms were explored by docking compounds 3 and 4 into the RNA tunnel of DENV2 NS5 RdRp. Both compounds initially exhibited favorable binding, forming hydrogen bonds with key residues, including Y607, N493, T606, G608, and T611 (Supplementary Table S8, Supplementary Fig. S1). Compound 4 exhibited more extensive interactions than compound 3, suggesting a more stable starting conformation (Supplementary Table S9, Supplementary Fig. S1). However, 300 ns MD simulations revealed dynamic and partially unstable binding. Compound 3 showed significant displacement toward the upper tunnel in one replicate, while compound 4 remained more centrally located (Supplementary Fig. S2A). Both compounds displayed fluctuating RMSD and hydrogen bond profiles (Supplementary Fig. S2B–C). Although transient contacts with residues G608, T606, and G662 persisted near the chlorine-substituted moiety, the unstable binding suggests that RdRp may not be the primary target.

Collectively, the in silico analyses identified DENV2 NS5 methyltransferase as the most plausible viral target of chlorinated biscoumarins, with compounds 3 and 4 showing strong predicted binding within the SAM pocket and forming stabilizing interactions with key catalytic residues. Although docking also suggested possible engagement with NS5 RdRp, subsequent molecular dynamics simulations revealed unstable, transient interactions that were inconsistent with high-affinity binding. These findings support NS5 MTase as the primary viral protein engaged by the compounds while indicating that RdRp is unlikely to be a major antiviral target.

### Lead compounds demonstrate broad-spectrum efficacy against all DENV serotypes and zikvselectivity

Based on the primary antiviral screening (Table 1), compounds 3, 4, and 8 were identified as the most active inhibitors of both DENV2 and ZIKV. These three compounds were therefore selected for cytotoxicity profiling across multiple human- and non-human–derived cell lines (Table 3, Supplementary Fig. S3). Cytotoxicity was assessed by treating cells with ten concentrations of each compound and quantifying cell viability using the MTS assay. Among the three candidates, compound 3 consistently exhibited the lowest cytotoxicity across all tested cell types, indicating a more favorable safety profile. In contrast, compound 4 showed increased cytotoxicity particularly in human-derived cell lines (A549, HepG2, and HEK293), while compound 8 displayed the highest cytotoxicity overall, irrespective of cell origin. The elevated toxicity of compound 8 substantially reduced its selectivity index, making it unsuitable for further development.

Because compounds 3 and 4 demonstrated both measurable antiviral activity and acceptable cytotoxicity profiles, they were chosen for expanded efficacy analyses. EC_50_ values for compounds 3 and 4 against all four DENV serotypes and ZIKV are presented in Table 4, supporting the claim of broad-spectrum antiviral activity (Table 4, Supplementary Fig. S4). Ten concentrations of each compound were applied to virus-infected Vero cells, and infectious viral titers were quantified by plaque assay. Both compounds yielded comparable EC_50_ values against all four DENV serotypes and ZIKV, consistent with the broad-spectrum inhibition observed in the primary screen.

To assess antiviral performance in human cellular environments, we further evaluated both compounds against DENV2 in three human-derived cell lines commonly used as permissive cells for studying DENV infection. Although antiviral efficacy was reduced in A549 and HEK293 cells (EC_50_ = 8.71–10.88 µM), compound 3 maintained a moderate selectivity index (SI = 7.37–8.79). Despite the recommended SI threshold of ≥ 10^16^, identifying the molecular target(s) of compound 3 remains valuable for structure–activity relationship analysis and future optimization. Compound 4, while structurally differentiated only by the position of a chlorine substituent, exhibited both lower potency and higher cytotoxicity compared with compound 3. Nevertheless, its inclusion in subsequent mechanistic studies provides an important opportunity to understand how subtle halogen positional changes influence antiviral activity.

In conclusion, compounds 3 and 4 demonstrated broad-spectrum antiviral activity across all four DENV serotypes and ZIKV, with EC_50_ values in the low micromolar range. Expanded cytotoxicity profiling revealed that compound 3 possessed the most favorable selectivity index and lowest cytotoxicity across diverse human cell lines, supporting its designation as the lead antiviral candidate. Although antiviral potency was lower in human-derived cell lines than in Vero cells, both compounds retained measurable activity, justifying their continued evaluation in downstream mechanistic studies.

**Table 3:** CC_50_s of compounds **3**, **4**, and **8**. Data represent the mean ± standard error of the means of three independent experiments (in µM).

CC_50_	Compounds		
	3	4	8
Vero	75.85 ± 3.25	66.97 ± 4.86	55.51 ± 5.92
HepG2	57.96 ± 2.92	38.50 ± 2.34	32.73 ± 1.75
A549	76.58 ± 4.74	53.75 ± 1.94	45.45 ± 3.66
HEK293	71.60 ± 3.67	44.35 ± 1.48	40.92 ± 1.62
BHK-21/DENV2 replicon	88.97 ± 7.59	69.68 ± 3.73	-

**Table 4:** EC_50_s of 2 selected compounds. Data represent the mean ± standard error of the means of three independent experiments (in µM). SI = selective index (CC_50_/EC_50_ ratio).

EC_50_	Compounds			
	3	SI	4	SI
DENV2 (Vero)	3.62 ± 0.67 (20.97)	20.97	4.62 ± 0.63 (14.50)	14.50
DENV2 (HepG2)	6.89 ± 0.97 (8.41)	8.41	5.08 ± 0.91 (7.57)	7.57
DENV2 (A549)	8.71 ± 1.01 (8.79)	8.79	10.82 ± 1.40 (4.97)	4.97
DENV2 (HEK293)	9.72 ± 1.18 (7.37)	7.37	10.88 ± 0.66 (4.08)	4.08
DENV1 (Vero)	3.20 ± 0.65 (23.72)	23.72	5.34 ± 0.63 (12.54)	12.54
DENV3 (Vero)	8.22 ± 0.67 (9.23)	9.23	11.80 ± 0.80 (5.67)	5.67
DENV4 (Vero)	2.85 ± 0.54 (26.63)	26.63	3.15 ± 0.39 (21.28)	21.28
ZIKV (Vero)	4.35 ± 0.25 (17.42)	17.42	4.19 ± 0.41 (15.98)	15.98

### Compounds 3 and 4 collectively inhibit DENV2 NS5 N7 and 2’-O methyltransferase activity

**Fig. 3:**
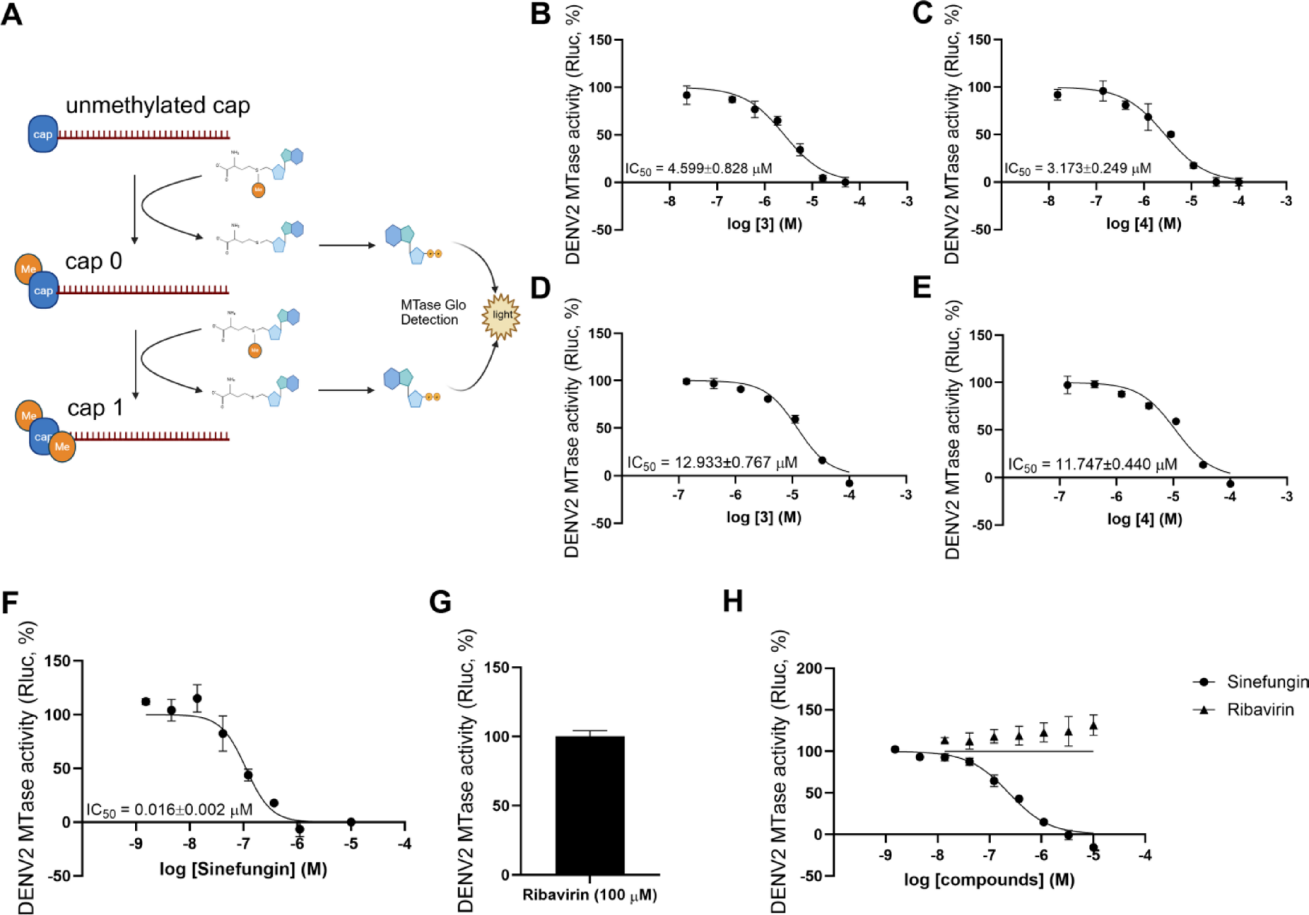
DENV2 Methyltransferase activity in the presence of compounds 3 and 4. (**A**) A schematic diagram demonstrates the 2-step capping mechanism. The DENV2 MTase, RNA substrates and eight concentrations of compounds were mixed in reaction buffer. SAM was added to start the reaction. The MTase reaction was incubated at 37°C for 30 min before adding MTase-Glo detection. The luminescence signals were determined and calculated for percentage of MTase activity. The RNA substrates were unmethylated cap and cap 0 for (**B**-**C**) total and (**D**-**E**) 2’-O methylation reactions, respectively. (**B**-**C**) demonstrated dose-dependent response curves of compounds 3 and 4 against the total DENV2 MTase activity, and (**D**-**E**) were IC_50_s against the 2’-O DENV2 MTase activity, respectively. The inhibitory concentration (IC_50_) was calculated from a non-linear regression curve-fit, and the concentration required for 50% luminescence reduction (IC_50_) was determined. Results were reported as the mean and standard error of the mean (SEM) from three independent experiments. (**F**-**H**) Sinefungin and ribavirin were used as positive and negative inhibitor controls, where (**F**-**G**) represented the total DENV2 MTase inhibition and (**H**) represented the 2’-O DENV2 MTase inhibition of both controls.

To evaluate whether NS5 methyltransferase (MTase) is a molecular target of compounds 3 and 4, we assessed their inhibitory effects on DENV2 MTase enzymatic activity. Flaviviral RNA capping proceeds through two sequential methylation steps: N-7 methylation of the guanine cap (cap 0), followed by 2′-O methylation of the ribose of the first nucleotide (cap 1) (Fig. 3A).

Both compounds inhibited total MTase activity, which reflects combined N-7 and 2′-O methylation. Compound 3 displayed an IC_50_ of 4.599 ± 0.828 µM, and compound 4 exhibited an IC_50_ of 3.173 ± 0.249 µM (Fig. 3B–C). To dissect their effects on the second methylation step, we next examined isolated 2′-O MTase activity using a cap 0 substrate. Compounds 3 and 4 inhibited 2′-O methylation with IC_50_ values of 12.933 ± 0.767 µM and 11.747 ± 0.440 µM, respectively (Fig. 3D–E), indicating that both methylation steps were affected, though with reduced potency for the 2′-O reaction.

For comparison, reference inhibitors, including sinefungin and ribavirin, were tested in parallel under identical experimental conditions, with concentration ranges adjusted to their known potency to validate each assay. The known SAM-competitive inhibitor sinefungin inhibited total MTase activity and isolated 2′-O activity with IC_50_ values of 0.016 ± 0.002 µM and 0.234 µM, respectively (Fig. 3F and H), consistent with its established mechanism of binding to the SAM-binding pocket. In contrast, compounds 3 and 4 were 287.43-fold and 198.31-fold less potent than sinefungin in the total MTase assay. Ribavirin, an RNA-dependent RNA polymerase inhibitor, exhibited no MTase inhibition and served as a negative control (Fig. 3G–H).

Overall, biochemical assays confirmed that compounds 3 and 4 inhibit both the N-7 and 2′-O methylation steps carried out by DENV2 NS5 methyltransferase, although with substantially lower potency than the SAM-competitive inhibitor sinefungin. While these findings support MTase as a mechanistic contributor to antiviral activity, the relatively modest inhibition suggests that MTase blockade alone does not fully account for the compounds’ cellular antiviral effects. Thus, additional viral or host pathways may participate in mediating the observed antiviral phenotype.

### **Serial passaging identifies compound-associated viral mutations that do not confer antiviral resistance**

**Fig. 4:**
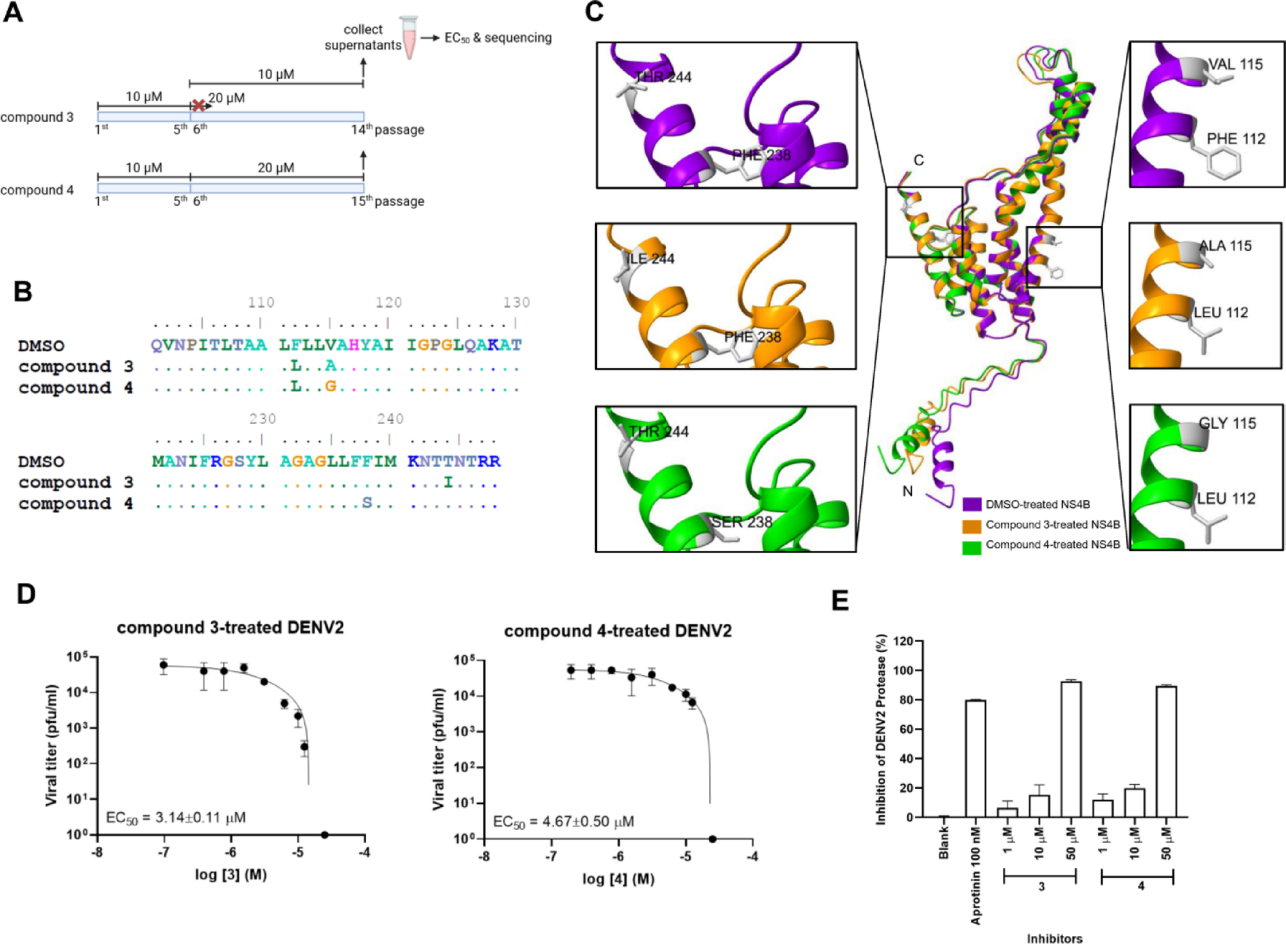
Effect of compounds on generation of mutation. (**A**) Diagram of experimental procedure Vero cells were infected with DENV2 in the presence of compounds or DMSO for 14–15 passages. Viruses recovered from passage 15th (DMSO- and compound 4-treated) and passage 14th (compound 3-treated) were used to determine for mutation by sequencing and EC_50_s. (**B**) Amino acid mutations found in NS4B protein (**C**) 3D structure of NS4B and amino acid substitutions found in protein. The protein structures were predicted by Alphafold 3 and the structure comparing was generated by ChimeraX. NS4B structures of the viruses obtained from the revertant mutants under treatment with DMSO, compound 3 and compound 4 were labeled with purple, orange and green colors, respectively. The point mutations in amino acid were mapped to transmembrane domain. (**D**) The EC_50_s of DENV2 recovered from the mutant assay. DENV2 mutants used in efficacy test were retrieved from passage 14th and 15th of compound 3 and 4 respectively. (**E**) In vitro DENV2 protease activity of compounds 3 and 4 at 1, 10, and 50 µM. Aprotinin (100 nM) was used as a positive inhibition control.

To investigate whether prolonged compound exposure could select for mutations in the viral NS5 MTase domain or other replication-associated regions, we performed serial passaging of DENV2 in the presence of compounds 3 or 4 (Fig. 4A). DMSO-treated cultures were included as controls. Virus-infected Vero cells were treated with 10 µM of either compound and incubated under standard culture conditions (5% CO₂, humidified chamber). Cultures were monitored daily, and supernatants were harvested once approximately 50% cytopathic effect (CPE) was observed. The collected viral supernatant was then used to infect fresh cells, and the corresponding compound was reintroduced at the beginning of each new passage. An identical approach was applied to both compounds.

After five passages, the compound concentration was increased to 20 µM. In the compound 3–treated group, no CPE was observed following dose escalation, so treatment was maintained at 10 µM for all subsequent passages. In contrast, compound 4 treatment produced CPE within 2–3 days after increasing the concentration to 20 µM, allowing propagation for an additional ten passages; however, the concentration of compound 4 could not be increased beyond 20 µM due to cytotoxicity. Viral particles from the 14th (compound 3) and 15th (compound 4) passages were isolated for EC_50_ determination and whole-genome sequencing.

Sequencing reads were aligned to the DENV2 genome obtained from DMSO-treated control virus passaged in parallel for 15 rounds, using the ClustalW algorithm in BioEdit to identify emergent mutations. Both compound-treated viruses exhibited clustered mutations in NS4B (Fig. 4B; Supplementary Fig. S5), with substitutions in transmembrane helices α5 (F112L, V115A/G) and α9 (F238S, T244I). Helix α9 is located within the C-terminal region of NS4B that mediates protein self-dimerization and contributes to the curvature of ER-derived spherules required for replication-complex formation. Structural predictions generated by AlphaFold 3 revealed no major conformational differences between native and mutant NS4B proteins (Fig. 4C).

Functionally, EC_50_ values of viruses recovered after serial passaging did not differ significantly from those of the parental strain (Fig. 4D), demonstrating that the observed mutations did not confer antiviral resistance. Instead, these substitutions likely represent indirect, host-interaction–dependent adaptive changes, a phenomenon previously described in flaviviruses and other RNA viruses during long-term culture under altered cellular conditions or mild selective pressure.

Protease inhibition assays showed that compounds 3 and 4 reduced NS2B/NS3 protease activity at 50 µM (Fig. 4E), suggesting a potential impact on viral polyprotein processing. However, their potency was approximately 50-fold lower than that of the known inhibitor aprotinin, making the protease an unlikely primary antiviral target. Although NS5 polymerase activity was not assessed in this study, the combined evidence of broad anti-flaviviral and anti-alphaviral activity reported for biscoumarins suggests that the principal antiviral effects may involve host-regulated rather than solely virus-encoded pathways.

Because compounds 3 and 4 are relatively new chemical entities, limited information is available regarding their cellular interaction profiles. Using the Chemical Similarity Ensemble Approach (SEA)^39^, we analyzed structurally similar compounds (≥ 95% Tanimoto similarity) deposited in PubChem and categorized predicted host targets into three groups: (1) proteins associated with viral translation/replication, such as PLK1 and HTT; (2) Inflammation-related proteins, including USP1 and NQO1; and (3) Mitochondrial or apoptotic regulators, such as caspase-6 (CASP6) and HTT (Supplementary Table in^17^. Additional predicted targets (e.g., ADAM17, PGE2 receptor, ALOX12) are linked to inflammatory or tissue-damage pathways. Although these predictions require experimental validation, they suggest that chlorinated biscoumarins may modulate host factors that indirectly influence viral replication, aligning with the mutation patterns observed during serial passaging.

Together, the serial passaging experiments revealed adaptive mutations arising in NS4B under compound treatment pressure, particularly within transmembrane helices linked to replication-complex formation. Despite their emergence, these mutations did not affect compound susceptibility, demonstrating that neither compound 3 nor 4 induced antiviral resistance. Combined with modest protease inhibition and absence of NS5 MTase mutations, the data suggest that the primary antiviral effects of biscoumarins are likely involve broader perturbations of replication-complex dynamics or host-dependent processes rather than direct inhibition of a single viral enzyme.

### Compounds 3 and 4 potently inhibit viral translation in DENV2-infected cells and DENV2 replicon cells

**Fig. 5:**
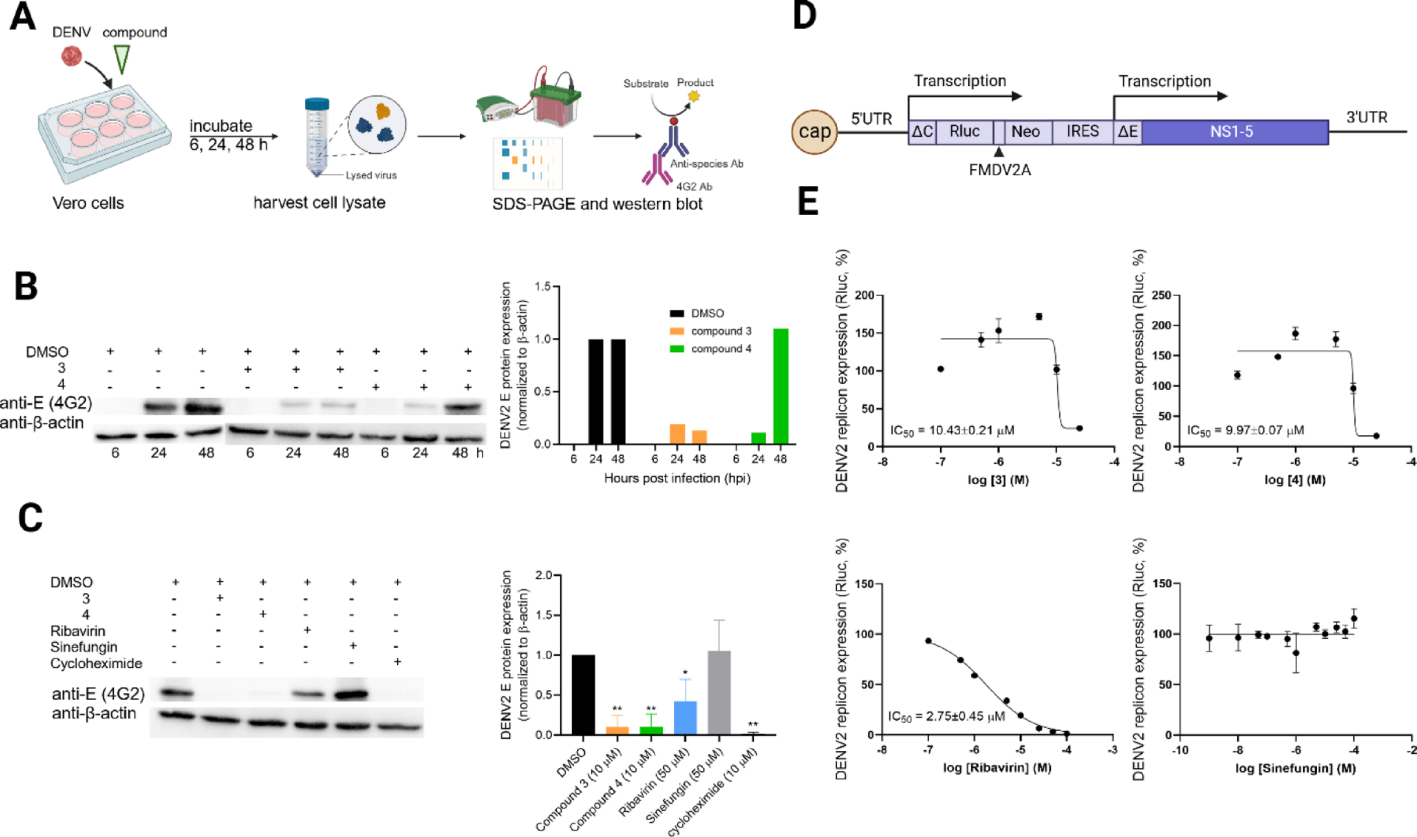
Inhibition of viral translation in western blot and replicon assays. (**A**) Schematic diagram and (**B**-**C**) western blotting results. Vero cells were infected with DENV2 in the present of compound and incubated for 6, 24 and 48 h. Cell lysates were collected and the proteins were extracted using NP-40 lysis buffer. The proteins were analyzed for viral protein expression using anti-E antibody (4G2) as a primary antibody. The β-actin was used as an internal control. (**A**-**B**) DENV2-infected Vero cells were treated with the compounds for 6, 24, and 48 h and the blot intensity was proportionated with DMSO-treated controls at respective time points. (**C**) The 24 h time points were further characterized with compounds 3, 4, sinefungin, ribavirin, and cycloheximide. Errors indicated standard deviations of two independent experiments. *, ** indicated the significant difference from DMSO treatment (unpaired *t*-test with Welch’s corrections) (**D**-**E**) Cap-dependent Rluc expression in DENV2 replicon. (**D**) the replicon construct showed Rluc expression driven by cap-dependent translation. (**E**) compounds were added at eight different concentrations and incubated for 1 day before quantifying the Rluc expression level. The nonlinear regression curves were plotted to identify half-maximal inhibitory concentration (IC_50_). Errors indicated standard deviations of three independent experiments.

We next examined whether the biscoumarin derivatives affect viral translation and RNA replication. Flaviviral translation occurs from a single open reading frame that produces a ~ 379-kDa polyprotein, which is subsequently cleaved into structural and non-structural proteins. To assess the impact of the compounds on translation and early polyprotein processing, we measured viral envelope (E) protein expression at 6, 24, and 48 h post-infection (hpi) (Fig. 5A–B). Compound 3 markedly reduced viral translation, with 81% and 89% inhibition at 24 and 48 hpi, respectively. Compound 4 showed 87% inhibition at 24 hpi, although the effect diminished by 48 hpi (Fig. 5B, Supplementary Fig. S6). When translation efficiency was evaluated at 24 hpi across all treatments, both compounds significantly suppressed E-protein expression, as detected using the 4G2 monoclonal antibody (Fig. 5C, Supplementary Fig. S6).

Interestingly, the known SAM-competitive methyltransferase inhibitor sinefungin did not substantially reduce viral translation in infected cells, whereas ribavirin—an inhibitor of viral RNA-dependent RNA polymerase (RdRp)—showed a stronger inhibitory effect. The superior translation inhibition produced by the biscoumarins compared with sinefungin suggests that their primary antiviral activity may involve cellular or host-regulated pathways rather than solely direct MTase inhibition.

To further evaluate cap-dependent translation and replication, we used a DENV2 subgenomic replicon in which the structural genes were replaced with a Renilla luciferase (Rluc) reporter (Fig. 5D). The replicon was stably maintained in BHK-21 cells, and Rluc expression served as a quantitative readout of ongoing translation from replicating RNA. Both compounds inhibited replicon translation in a dose-dependent manner, with IC_50_ values of 10.43 ± 0.21 µM for compound 3 and 9.97 ± 0.07 µM for compound 4 (Fig. 5E).

As expected, ribavirin strongly suppressed replicon activity (IC_50_ = 2.75 ± 0.45 µM), consistent with its dual effects on RdRp inhibition and indirect interference with SAM-dependent processes through the ribavirin triphosphate metabolite. In contrast, sinefungin did not significantly inhibit replicon translation or replication, consistent with its known activity requiring binding to the SAM-binding site within the NS3–NS5–SLA elongation complex^2^, a configuration not fully engaged during replicon-driven translation.

Overall, the replicon data demonstrate that biscoumarins inhibit viral translation and replication more effectively than sinefungin but less potently than ribavirin. Taken together with the translation assays in infected cells, these results suggest that the primary antiviral effects of compounds 3 and 4 extend beyond classical MTase inhibition at the SAM-binding site and may involve disruption of host-dependent pathways essential for flaviviral translation and RNA synthesis.

In summary, both compounds significantly suppressed viral translation in DENV2-infected cells and in a subgenomic replicon system, with reductions in viral protein expression exceeding those achieved with sinefungin. These findings indicate that translation and early RNA replication processes are major points of antiviral intervention. The stronger inhibitory effects observed in cell-based systems compared with MTase enzymatic assays suggest that the compounds likely exert their antiviral activity through a combination of partial MTase inhibition and interference with additional viral or host factors involved in translation and RNA synthesis.

### Time-course study and replicon system reveal dynamic, reversible Inhibition of viral replication

**Fig. 6:**
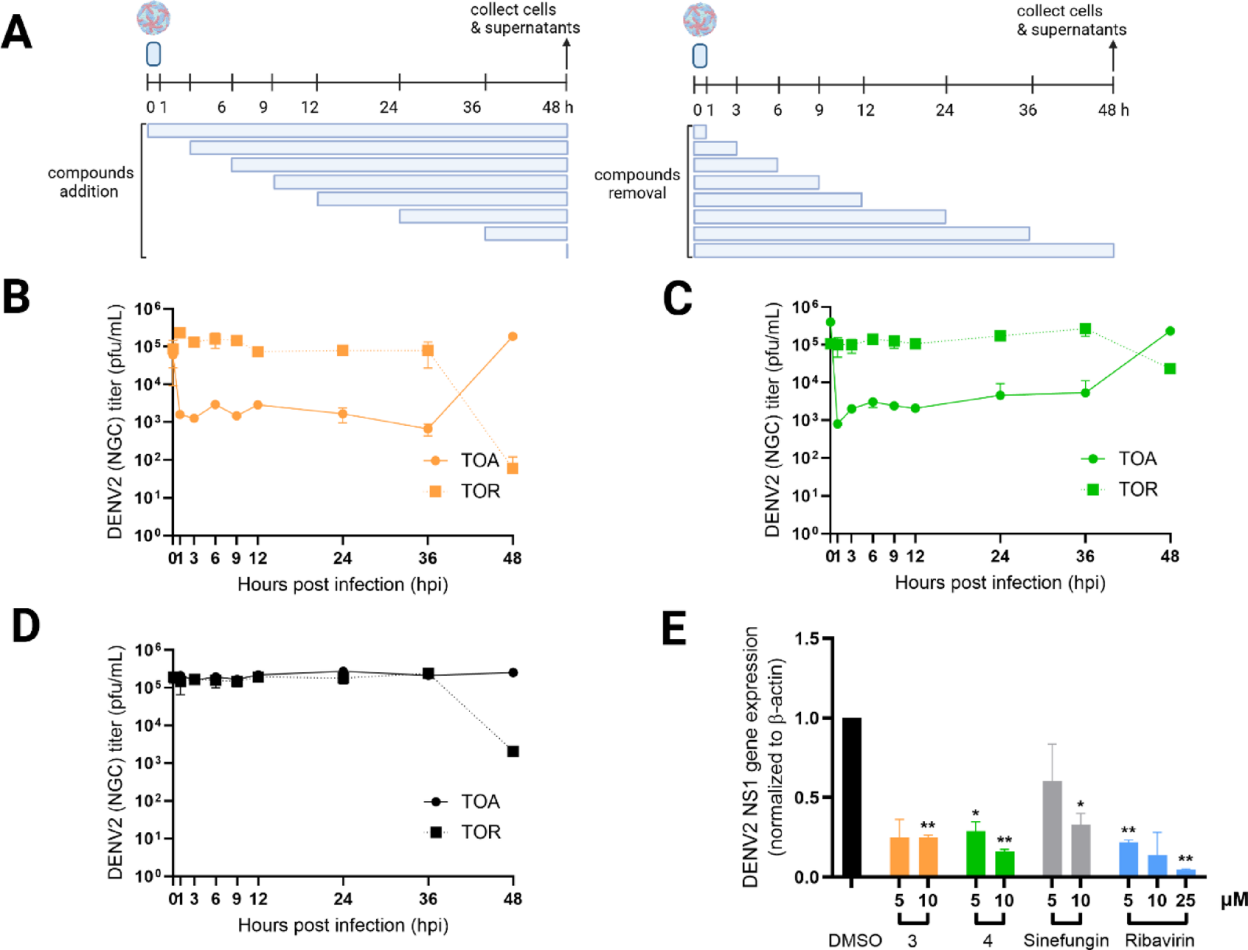
Viral replication analysis. (**A**) Schematic diagram of time-of-addition and time-of-removal of (**B**) compound **3** and (**C**) compound **4**. (**D**) DMSO was used as no inhibition control. (**E**) The level of NS1 gene expression in BHK-21/DENV2 replicon cells incubated with compounds for 24 h. The replicon cells were incubated with compounds for 24 h. The replicon RNA was extracted and quantified for NS1 expression level by RT-qPCR. The NS1 expression levels were normalized to β-actin and calculated as fold expression compared to no inhibitor (DMSO) control. Errors indicated standard deviations of three independent experiments. *,** referred to the significant levels of *p* < 0.05 and *p* < 0.01, respectively using unpaired *t*-test with Welch’s corrections.

To further examine how the biscoumarin compounds affect the kinetics of the viral replication cycle, we performed time-of-addition and time-of-removal experiments (Fig. 6A). Compounds 3 and 4 were added to DENV2-infected Vero cells at multiple time points or removed after defined periods to assess their temporal requirement for antiviral activity. Both compounds inhibited viral replication regardless of when they were introduced, including when added as late as 36 h post-infection (hpi) (Fig. 6B–C). Conversely, removal of either compound at any time point led to an immediate restoration of viral replication, even when the compounds had been present for extended periods. DMSO-treated, DENV2-infected cells served as mock controls (Fig. 6D).

This inhibition profile suggests that the compounds act directly on ongoing viral replication processes rather than on early entry or translation initiation events. The rapid recovery of viral replication upon compound withdrawal further indicates that compound–target interactions are highly dynamic and transient, requiring the continuous presence of the compound to sustain inhibition.

We next evaluated the effect of compounds 3 and 4 in a BHK-21 DENV2 replicon system, in which Renilla luciferase expression reflects active translation of replicating RNA. After 24 h of compound exposure, replicon RNA levels—measured by NS1 gene expression—were significantly reduced (Fig. 6E), confirming that the compounds impair RNA synthesis. Consistent with earlier translation assays, the biscoumarins displayed stronger inhibitory activity than sinefungin but remained less potent than ribavirin.

Together, these results demonstrate that compounds 3 and 4 effectively suppress viral replication even when administered during late stages of infection. Continuous compound presence is necessary to maintain inhibition, indicating that their antiviral activity likely targets dynamic components of the replication machinery or host factors that support viral RNA synthesis. Integrating these findings with earlier data, compounds 3 and 4 consistently inhibit viral protein expression, RNA replication, and infectious virus production without selecting for resistance-associated mutations during extended exposure.

## Discussion

Coumarins and their derivatives are well known for their diverse biological activities, including antiviral effects across multiple virus families. In this study, we identified chlorinated bis-4-hydroxycoumarin derivatives as potent inhibitors of dengue virus (DENV) replication. Structure–activity relationship (SAR) analysis demonstrated that specific substitutions on the phenyl ring—particularly chlorine at the R2 or R3 positions—substantially enhanced antiviral potency while maintaining comparable, mild cytotoxicity. Among the synthesized analogs, compounds 3 and 4 emerged as the strongest candidates, displaying broad-spectrum inhibition across all DENV serotypes and Zika virus (ZIKV). In contrast to prior work focusing on chikungunya virus efficacy and pharmacokinetics, the present study emphasizes mechanistic target identification in flavivirus-infected cell systems, thereby extending the understanding of biscoumarin antiviral activity.

Chlorine substitutions at the 3- or 4-position were superior to other halogens. Substituting chlorine with bromine or fluorine (compounds 5 and 6) reduced antiviral activity, especially against ZIKV, and replacing 3-Cl with 3-NO₂ or 3-Br (compounds 9–12) similarly attenuated inhibition. Molecular docking suggested that residue R84 forms a stabilizing alkyl–π interaction with the 3- or 4-Cl substituent, while surrounding hydrophobic residues (S56, C82, G83, G85, G86) form a tight pocket that may disfavor the larger Br atom and provide weaker interactions with the electronegative F atom. These structural insights explain the observed SAR trend. Compound 3 exhibited a higher selectivity index (SI) and lower cytotoxicity than compound 4 across multiple human-derived cell lines, making it the more favorable antiviral lead.

Initial docking studies predicted that DENV NS5 methyltransferase (MTase) could be a potential target; however, biochemical assays showed that compounds 3 and 4 inhibited MTase activity far less effectively than sinefungin, by 287-fold and 198-fold, respectively. Although NS5 methyltransferase was identified as a plausible viral target, the modest biochemical inhibition relative to sinefungin and the strong suppression of viral translation in cell-based assays indicate that MTase inhibition alone does not fully account for the antiviral phenotype.

We also examined binding to the NS5 RNA-dependent RNA polymerase (RdRp). Docking suggested potential interactions near the RNA tunnel, but 300-ns molecular dynamics (MD) simulations revealed unstable and highly dynamic contacts, particularly for compound 3. RMSD fluctuations and rapidly forming/dissolving hydrogen bonds indicated that neither compound achieves a stable binding mode in the RdRp active tunnel. This contrasts with other coumarin derivatives that stably engage HCV NS5B RdRp^18^, suggesting that NS5 polymerase is not the principal target of bis-4-hydroxycoumarins in DENV.

Beyond flaviviruses, compounds 3 and 4 also inhibited chikungunya virus (CHIKV) with EC_50_ values of 2.85 ± 0.42 µM and 3.08 ± 0.45 µM, respectively^17^ indicating broader activity spanning both flaviviruses and alphaviruses. Their antiviral potency varied among cell lines, possibly reflecting differences in host dependency factors or intracellular accumulation. In vivo toxicity assessments further indicated acceptable pharmacokinetic profiles. For CHIKV, nsP1 MTase was a predicted viral target, while host-based targets remained less defined. Using the Chemical Similarity Ensemble Approach (SEA), we identified several potential cellular targets for biscoumarins, including proteins involved in viral translation/replication (PLK1, HTT), inflammatory responses (USP1, NQO1), and mitochondrial pathways (caspase-6). Additional predicted interactions with ADAM17, the PGE₂ receptor, and ALOX12 suggest potential immunomodulatory effects. Although these predictions require experimental validation, they offer valuable hypotheses for future mechanistic studies.

Serial passaging experiments revealed clusters of mutations in transmembrane helices α5 (F112L, V115A/G) and α9 (F238S, T244I) of NS4B, a protein critical for membrane remodeling and replication-complex formation. The α9 helix, in particular, contributes to NS4B dimerization and the curvature of ER-derived spherules. Despite these mutations, the resulting viruses exhibited EC_50_ values indistinguishable from the parental strain, indicating that the substitutions did not confer resistance. Instead, they likely represent adaptive responses to replication stress or altered host–virus interactions under compound pressure. This aligns with observations that replication-complex components such as NS4B frequently accumulate compensatory—not resistance—mutations when NS5 function or replication-complex dynamics are perturbed.

Several studies, including those on clinical trial drugs like JNJ-A07, have described NS4B as a hotspot for resistance mutations in flavivirus replication complexes^19^. The absence of mutations in the NS5 MTase domain further supports the idea that this enzymatic region is structurally constrained and less tolerant of adaptive changes.

Importantly, biscoumarins demonstrated strong inhibition of viral translation in cell-based assays. Compound 3 suppressed envelope (E) protein levels by up to 89%, and both compounds inhibited cap-dependent translation in replicon assays with IC_50_ values near 10 µM. Translation inhibition was substantially stronger than MTase or protease inhibition, suggesting that the primary antiviral effects occur through disruption of viral translation or replication-complex assembly, potentially involving allosteric modulation of NS5 or perturbation of host factors essential for RNA synthesis. Sinefungin did not significantly inhibit cellular translation, supporting the hypothesis that biscoumarins act through mechanisms beyond classical SAM-competitive inhibition.

Time-of-addition and time-of-removal studies further revealed that biscoumarins inhibit replication even when added at late time points (up to 36 hpi), and that viral replication resumes rapidly upon compound withdrawal. This behavior suggests dynamic and reversible interactions with their molecular targets—properties desirable in antiviral leads. The absence of resistance-conferring mutations following long-term serial passaging, together with reversible inhibition observed in time-of-addition and removal experiments, supports a model in which chlorinated biscoumarins perturb dynamic replication-complex processes and host-dependent pathways.

Despite these promising findings, several challenges remain. Optimization of pharmacokinetics, bioavailability, and in vivo stability will be essential for therapeutic development. Additionally, combination therapy—particularly with RdRp inhibitors or NS4B-targeting agents—may enhance antiviral efficacy and reduce the likelihood of resistance emergence.

Overall, chlorinated bis-4-hydroxycoumarins represent a promising new scaffold for antiviral development. Their broad-spectrum anti-flaviviral and anti-alphaviral activity, combined with their ability to inhibit viral translation and replication through multi-target or host-modulating mechanisms, provides a compelling foundation for further medicinal chemistry optimization. Future work should focus on refining structural features, elucidating host-factor interactions, and validating efficacy in vivo. These compounds may ultimately contribute to the development of next-generation antiviral strategies targeting DENV, ZIKV, and other clinically significant arboviruses.

## Methods

### Compound synthesis

Twelve biscoumarin analogs **1**–**12** were synthesized as previously described^12^. The compounds were characterized by ^1^H-NMR, ^13^C-NMR, and HPLC analysis. The compounds were dissolved in DMSO (PanReac AppliChem, Hesse, Germany) at 50 mM stock and stored in aliquots at -20 °C until use.

Sinefungin was purchased from Abcam, Cambridge, UK, and ribavirin from Bio Basic, Ontario, Canada.

### Cells and viruses

Vero (ATCC^®^CCL-81), LLC/MK2 (ATCC^®^CCL-7), C6/36 (ATCC^®^CRL-1660), HepG2 (ATCC^®^HB-8065), HEK-293 (ATCC^®^CRL-1573) and A549 (ATCC^®^CCL-185) cells were maintained as previously described^20–22^.

Reference strains of DENV1 (16007), DENV2 (NGC), DENV3 (16562), DENV4 (c0036), and ZIKV (SV0010/15) were propagated in C6/36 cells as previously described^20,21^.

### Primary screening

The efficacy and cytotoxicity screening of compounds were tested as previously described^20,21,23^. Vero cells were selected as the primary cell line for antiviral screening, plaque assays, and time-course studies due to their high permissiveness to flavivirus infection and established use in dengue and Zika virus research. DENV serotype 2 (DENV2) was selected as the representative strain for mechanistic studies because secondary infection with DENV2 is consistently associated with severe clinical outcomes, making it a stringent benchmark for antiviral evaluation. Primary antiviral screening was performed at a single compound concentration of 10 µM to reliably identify compounds with micromolar-range antiviral potency while minimizing false-negative exclusion, following established antiviral discovery protocols. Vero cells (5 × 10^4^) were seeded in a 24-well plate and incubated at 37 °C under 5% CO_2_ overnight. Cells were infected with DENV2, or ZIKV at a multiplicity of infection (MOI) of 0.1 and a compound at 10 µM was added during the infection. DMSO at 1% was used as no inhibition control. Cultures were incubated for 1 h with gentle rocking every 15 min. Cells were washed with PBS, maintenance medium supplemented with 1% FBS, 100 I.U./mL penicillin and 100 µg/mL streptomycin, 10 mM HEPES was added in a presence of a compound. Cells were incubated at 37 °C with 5% CO_2_ for 3 and 2 days in DENV2 and ZIKV infections, respectively. Supernatants were collected to determine viral titers by plaque titration assay. Results were reported from three replicates of one experiment.

Cytotoxicity of the compounds was accessed in parallel with the viral inhibition. Vero cells (1 × 10^4^) were seeded in 96-well plate and incubated at 37 °C under 5% CO_2_ overnight. Compounds were added and incubated for 2 days. DMSO at 1% was used as a mock treatment. Cytotoxicity was measured using CellTiter 96® AQueous One Solution Cell Proliferation Assay (MTS) kit (Promega, Wisconsin-Madison, USA) according to the manufacturer’s instruction and analyzed by spectrophotometry at *A*_490 nm_. Results were reported from three replicates of one experiment.

### Efficacies and cytotoxicities of the lead compounds

For EC50 determination, ten concentrations (0.1–50 µM, two-fold serial dilutions) were tested, and selectivity indices (SI) were calculated as CC50/EC50. Vero cells (5 × 10^4^) were seeded in 24-well plate and incubated at 37 °C under 5% CO_2_ overnight. Cells were infected with DENV1, DENV2, DENV3, DENV4, or ZIKV at MOI of 0.1 and the selected compounds were analyzed for an effective concentration (EC_50_). Each compound was serially diluted by two-fold dilution to ten different concentrations (0.1 to 50 µM) and DMSO at 1% was used as no inhibition control. Cultures were incubated for 1 h with gentle rocking every 15 min. Cells were washed with PBS and maintenance medium was added in the presence of a compound. Cells were incubated at 37 °C with 5% CO_2_ for 3 days and 2 days for DENV and ZIKV, respectively. Supernatants were collected to determine viral titers by plaque titration assay. The efficacy of each compound was calculated from non-linear regression curve-fit and the concentration required for 50% viral titer reduction (EC_50_) was determined using PRISM version 9 (GraphPad Software, La Jolla, CA, USA). Results were reported as mean and standard error of mean (SEM) from three independent experiments.Cytotoxicity of the compounds was accessed in parallel with the viral inhibition. Vero cells (1 × 10^4^) were seeded in 96-well plate and incubated at 37 °C under 5% CO_2_ overnight. Compounds were added and incubated for 2 days. DMSO at 1% was used as mock treatment. Cytotoxicity was measured using an MTS kit. The cytotoxicity concentration (CC_50_) of each compound was calculated from non-linear regression curve-fit and the concentration required for 50% cell death (CC_50_) was determined. Results were reported as mean and standard error of mean (SEM) from three independent experiments.

The selected compounds were evaluated for DENV2 inhibition and cytotoxicity in human cells, including HepG2, A549 and HEK293 using the same conditions as described above to confirm broad-spectrum efficacy across distinct cell types.

### Cell-based time-of-addition and time-of-removal assay

The possible target of the compound was initially screened by time-of-addition (TOA) and time-of-removal (TOR) assay. Vero cells (5 × 10^4^) were seeded in 24-well plate and incubated as described. Cells were infected with DENV2 at MOI of 1 for 1 h with gently rocking every 15 min. For TOA, the compound at 10 µM was added at various time points; 0, 1, 3, 6, 9, 12, 24, and 48 h post-infection (hpi). For TOR, the compound was added from the start, then removed and replaced with maintenance medium at each time point as TOA. DMSO at 1% was used as no inhibition control. Supernatants were collected at 48 hpi to determine viral titers by plaque titration assay. Results were confirmed by three independent experiments.

### QSAR study

Biscoumarin derivatives were tested for their antiviral activity in DENV2 and ZIKV cell-based assays. To identify common structural features associated with activity in both assays, a quantitative structure–activity relationship (QSAR) analysis was performed. In this study, the percentage inhibition of biscoumarin derivatives from both assays was used as the dependent variable, transformed to log[% inhibition] for QSAR model construction. The molecular descriptors of biscoumarin derivatives were generated using the Material Studio program^24^. Twenty-three molecular descriptors, including steric, electrostatics, and spatial descriptors, are listed in supplementary Table S3. These descriptors were standardized to eliminate differences in scales and units. Genetic function approximation-multiple linear regression (GFA-MLR) was employed using the Material Studio program. For the QSAR-Machine learning (ML) models, all molecular descriptors were selected based on their importance as determined by the Gini Importance (GI). GI is a concept used in machine learning, specifically in decision tree algorithms like Random Forests, and it refers to the importance of a feature in predicting the outcome of a model. In this study, QSAR-ML models, including Random Forest (RF) and Gradient Boosting Regression (GBR), were developed and compared to identify the most effective model.

### In Silico pan-docking and molecular dynamics simulation

The crystal structures of DENV2 proteins used in this study are envelope protein (1OKE), NS2/3 protease (2FOM^25^, NS3 helicase (2BHR^26^, NS5 RdRp (6IZX^27^, and NS5 MTase (3EVG^28^, which is considered as the protein receptors for ligand binding prediction using the molecular docking method. The native inhibitors for E protein, NS2B/NS3pro, NS3 helicase, NS5 MTase, and NS5 RdRp were 3-100-22^29^, SYC-1307^30^, ATP^31^, Sinefungin^32^, and NITD-107^33^, respectively, and were used as reference data for the molecular docking study, similar to previously report^34^. The protein structures were prepared as receptors following standard protocols^35^. Native inhibitors and selected ligands, chosen based on their antiviral efficacy, were constructed and optimized using GaussView 6 and Gaussian 16 with the B3LYP/6–31 g* basis set^36^. A molecular docking was performed on each target using AutoDock Vina 1.2.3^37–39^ to predict the binding pose and energy of each compound with 10 replications. Subsequently, the top pose from each run was chosen to compare with the binding energy of the native inhibitor of each viral target to identify the promising target for the compound.

Molecular dynamics (MD) simulations were performed for compounds 3 and 4 in complex with DENV2 RdRp at the RNA tunnel site, using binding poses obtained from molecular docking. Each complex was subjected to 300 ns MD simulations using the AMBER24 software suite. System preparation employed tLEaP with ff19SB for the protein and GAFF2 for ligands. Protonation states were assigned at pH 7.4 via PDB2PQR. Complexes were solvated in a TIP3P water box, neutralized with Cl^-^ ions, and simulated under periodic boundary conditions in the NPT ensemble (310 K, 1 atm). After two-step energy minimization and restrained equilibration, unrestrained production runs were conducted. Electrostatics were treated with PME, and SHAKE constraints were applied to hydrogen bonds. CPPTRAJ was used to analyze RMSD and hydrogen bonds. Binding free energy was estimated using MM-PBSA to evaluate key stabilizing interactions.

### In vitro methyltransferase assay

The MTase assay for compound inhibition testing was adapted and optimized as previously described^40,41^. In brief, the DENV2 MTase was expressed and purified from bacterial expression system. The RNA substrate was the first 200-nucleotide of DENV2 RNA sequence in vitro transcribed from the recombinant pET32a plasmid using T7 promoter (Genscript, Piscataway, NJ, USA). The RNA was capped using vaccinia capping system (New England Biolabs, Ipswich, MA, USA) in the absence of SAM for the N7 substrate, and in the presence of SAM for the 2’-O MTase substrate. The 10 µL of the enzyme complex (200 ng (169 nM) DENV2 MTase, 1.25 µM capped RNA substrate, and 10× MTase-Glo reagent (Promega, Madison, WI, USA) in 4× reaction buffer (80 mM Tris–HCl pH 8.0, 200 mM NaCl, 4 mM EDTA, 12 mM MgCl_2_, 0.4 mg/mL BSA, and 4 mM DTT)) was mixed with 5 µL of eight concentrations of compound (0.046 to 100 µM) and incubated at room temperature for 10 min. Sinefungin and DMSO were used as positive and negative inhibition controls. Subsequently, 10 µL of 2.5 µM S-adenosylmethionine (SAM) was added and incubated at 37 °C for 30 min. After incubating, 25 µL of MTase-Glo detection solution was added and incubated at room temperature for 60 min. The luminescence was measured using a VICTORTM X3 2030 Multilabel Reader (Perkin Elmer, Waltham, MA, USA). The inhibition of each compound was calculated from a non-linear regression curve fit, and the concentration required for 50% luminescence reduction (IC_50_) was determined. Results were reported as mean and standard error of mean (SEM) from three independent experiments.

### In vitro protease assay

#### Protein expression

The gene for the DENV2 NS2B-NS3 fusion protein was synthesized and cloned into the NdeI and HindIII of pET24a (Biomatik). The sequence of the expression construct with an N-terminal His_8_ tag and tobacco etch virus (TEV) protease cleavage site is given at the end of this paragraph. The sequence of the protein after TEV cleavage is identical to a previously reported construct. (cite PMID: 24164286) The expression plasmid was transformed into *Escherichia coli* Tuner (DE3) and selected on an LB agar plate containing 50 µg/mL kanamycin. A single colony was picked and inoculated into 100 mL of Terrific Broth containing kanamycin. The overnight culture was seeded into the Terrific Broth at a concentration of 3% and grown at 37 °C with shaking. IPTG was added to 0.1 mM when the OD600 reached 0.6 to induce protein expression. The cells were incubated with shaking at 37 °C for 6 h. The cells were harvested by centrifugation at 6,000 x g.

#### Protein purification

The *E. coli* cell pellet was resuspended and lysed in a buffer containing 20 mM HEPES pH 7.5, 150 mM NaCl, 25 mM imidazole by sonication on ice. The lysate was clarified by centrifugation at 40,000 x g for 30 min at 4 °C. The recombinant protein in the supernatant was then purified by Ni-NTA affinity chromatography using the imidazole gradient from 50 to 250 mM. Fractions containing pure protein, as examined by SDS PAGE, were pooled, and TEV protease was added. The mixture was dialyzed against 20 mM HEPES pH 7.5, 150 mM NaCl, 25 mM imidazole overnight at 4 °C. The cleaved protein was applied to a Ni-NTA column, and the DENV2 NS2B-NS3 protease was collected in the flow-through. The purified protein was dialyzed against 20 mM Tris pH 7.0, overnight at 4 °C.

#### In vitro protease assay

The enzymatic activity of DENV2 NS2B-NS3 protease was assessed using a fluorogenic peptide substrate, Bz-nleKRR-AMC (Biomatik), which releases 7-amino-4-methylcoumarin (AMC) upon cleavage. Each reaction was carried out in a final volume of 100 µL in black 96-well plates. The final reaction conditions were 50 mM Tris pH 7.5, 10 mM NaCl, 20% glycerol, 1 mM CHAPS, 1 µM DENV2 NS2B-NS3 protease, and 60 µM Bz-nleKRR-AMC substrate. The reactions were incubated at 25 °C while fluorescence was measured at an excitation wavelength of 380 nm and emission at 460 nm using a BioTek Synergy H1 microplate reader. Readings were recorded every 10 s over a 10-minute period. The rate of the reaction was used as the activity of the enzyme. The half-maximal inhibitory concentration (IC_50_) values were determined by incubating 1 µM enzyme, 60 µM substrate, and varying concentrations of the inhibitors. A plot of percentage activity versus the inhibitor concentration was generated using the GraphPad Prism 8 software.

### Replicon Inhibition assay

The BHK-21 cells stably expressed DENV2 replicon (BHK-21/DENV2) were maintained in MEM supplemented with 5% FBS and 300 µg/ml geneticin (Bio Basic, Ontario, Canada)^42^. The replicon cells (1 × 10^4^) were seeded in 96-well plate and incubated overnight. The medium was removed, and a maintenance medium containing eight different concentrations of compounds (0.1 to 100 µM) was added and incubated for 1 day. The ribavirin and sinefungin were used as positive and negative inhibition controls, respectively. The replicon inhibition was detected by luminescence signal using *Renilla* luciferase assay system (Promega, Medison, WI, USA) according to the manufacturer’s instruction. Briefly, the medium was removed, and the cells were washed with PBS. The lysis buffer was added and the plate was shaken for 15 min. The substrate was added, and the luminescence was measured using a VICTORTM X3 2030 Multilabel Reader (Perkin Elmer, Waltham, MA, USA). The inhibition of each compound was calculated from a non-linear regression curve fit, and the concentration required for 50% luminescence reduction (IC_50_) was determined. Results were reported as mean and standard error of mean (SEM) from three independent experiments.

The replicon replication was quantified by RT-qPCR. The replicon cells (5 × 10^4^) were seeded in a 24-well plate and incubated overnight. The medium was removed, and a maintenance medium containing the selected compounds, DMSO, ribavirin, or sinefungin was added to the cells and incubated for 1 day. Total RNAs were extracted using RNeasy mini kit according to manufacturer’s protocol (Qiagen, Hilden, Germany) and quantified by RT-qPCR for NS1 using a Power SYBR^®^ Green RNA-to-CT™ 1-Step kit (Applied Biosystems™, Waltham, MA, USA) with a Step-OnePlus Real-Time PCR System ABI 7500 (Applied Biosystems™, Waltham, MA, USA)^20^. The β-actin gene was used as an internal control^43^.

### Western blotting

Vero cells (5 × 10^4^) were seeded in 24-well plate and incubated at 37 °C under 5% CO_2_ overnight. Cells were infected with DENV2 at MOI of 1, and the selected compounds or DMSO were added. The ribavirin and sinefungin were used as NS5 RdRp and NS5 MTase inhibitor, respectively. Cycloheximide and DMSO were used as protein translation inhibitor and negative control, respectively. Cells were incubated for 6, 24, and 48 h. Cell lysates were collected and proceeded to Western blotting as previously described^44^. Briefly, the infected cells were lysed using NP-40 lysis and centrifuged at 12,000 g for 20 min to obtain the cell lysate. Cell lysates were subjected to SDS-PAGE under nonreducing conditions for DENV E protein. The 4G2 monoclonal antibody (HB-112, ATCC, Manassas, VA, USA) was used as the primary. Mouse anti-actin antibody (Biolegend, San Diago, CA, USA) was used as a loading control. HRP-conjugated goat anti-mouse IgG antibody was used as a secondary antibody (Biolegend, San Diago, CA, USA). The chemiluminescence signals were developed using Immobilon Classico Western HRP substrate (Merck, Darmstadt, Germany) using Bio-Rad ChemiDoc XRS + system (Bio-Rad, Hercules, CA, USA). Protein band intensities were quantified using ImageJ software version 1.54 g.

The selected time point was chosen for further experiments. Vero cells were prepared and infected with DENV2 as previously described. Selected compounds or DMSO were added. The ribavirin, sinefungin, and cycloheximide were used as controls. Cells were incubated for indicated hours and cell lysated was collected for protein quantification as previously described.

### Generation of revertant mutant

Vero cells (5 × 10^4^) were seeded in 24-well plate and incubated at 37 °C under 5% CO_2_ overnight. Cells were infected with DENV2 at MOI of 0.1.Compound 3 and 4 at 10 µM was added individually in parallel during and after the infection. DMSO was used as a control subpassage in parallel with the compound treatment. The infected cells were incubated until CPE was observed over 50% or up to 7 days. The supernatants were collected for a subsequent round of infection. The compounds were added to maintain the concentration throughout the study. After 5 passages, the concentration of the compound 4 increased to 20 µM and maintained at this concentration until the passage of 15. The compound 3 treatment was maintained at 10 µM throughout the 14 passages of compound treatment. Supernatants of the 14th passage with compound 3 and the 15th passage of compound 4 were collected for mutation analysis by plaque reduction assay and whole genome sequencing. Briefly, the DNA libraries were prepared by Ligation Sequencing gDNA - Native barcoding Kit 24 V14 (SQK-NBD 114.24) and then sequenced by MinION Mk1C sequencer with the R10.4.1 flow cell (Oxford Nanopore Technologies, UK).

### Nanopore data processing and analysis

Raw sequencing data in FAST5 format were basecalled using the Guppy basecaller v6.0.1 (Oxford Nanopore Technologies) with the super-accuracy (SUP) model to generate high-quality FASTQ reads. Quality assessment of the reads was performed using NanoPlot v1.20. Demultiplexing and adapter trimming were carried out using the integrated tools within MinKNOW v24.14 (Oxford Nanopore Technologies). The quality and distribution of the demultiplexed reads were evaluated using SeqStats (https://github.com/clwgg/seqstats). High-quality reads were then aligned to the reference genome of dengue virus serotype 2 (NC_001474.2) using Minimap2. The BAM files were visualized in the Integrative Genomics Viewer (IGV) to inspect mapping quality and genome coverage. Consensus sequences were generated from the alignments using iVar consensus (https://github.com/andersen-lab/ivar). Finally, the consensus genomes from all strains were aligned to the dengue virus reference genome (NC_001474.2) using ClustalW within BioEdit software v5.0.9 to identify mutations and sequence variations among the strains.

## Supplementary Information

Below is the link to the electronic supplementary material.

Supplementary Material 1

## Data Availability

The datasets used and/or analyzed during the current study are available from the corresponding author on reasonable request.

## Declarations

### Generative AI and AI-assisted technologies in the writing process

During the preparation of this work, the author(s) used ChatGPT, Gemini, and writefull in order to improve the academic English language. After using this tool/service, the author(s) reviewed and edited the content as needed and take(s) full responsibility for the content of the publication.
