# Supplementary material for: Chlorinated bis-4-hydroxycoumarins suppress flavivirus replication by inhibiting dengue virus type 2 translation and replication
Source: Sci Rep. 2026 Jan 16;16:5300. doi: 10.1038/s41598-026-35654-8 (PMC12881426; doi:10.1038/s41598-026-35654-8)
Supplement: Supplementary file 1 — Supplementary Material 1 [file 41598_2026_35654_MOESM1_ESM.docx]

**Supplementary Table S1** Primary screening results of DENV2 inhibition

| No. | Substitution (R) | DENV2 titer (pfu/ml) | | | Viral Inhibition (%) |
| --- | --- | --- | --- | --- | --- |
|  |  | R1 | R2 | R3 |  |
| DMSO control | - | 4.2x10^4^ | 4.2x10^4^ | 6.6x10^4^ | 0.00±22.63 |
| 2 | 2-Cl | 2x10^3^ | 3x10^3^ | 2.4x10^3^ | 95.07±0.82 |
| 3 | 3-Cl | 1.6x10^2^ | 1.8x10^2^ | 3.2x10^2^ | 99.56±0.14 |
| 4 | 4-Cl | 3.6x10^2^ | 3.4x10^2^ | 4x10^2^ | 99.27±0.05 |
| 5 | 4-F | 5.8x10^4^ | 6.6x10^4^ | 4.6x10^4^ | −13.33±16.44 |
| 6 | 4-Br | 1x10^3^ | 1.6x10^3^ | 1x10^3^ | 97.60±0.57 |
| 7 | 2,4-diCl | 1.6x10^3^ | 1.4x10^3^ | 1.6x10^3^ | 96.93±0.19 |
| 10 | 3-Br, 4-OH | 3.8x10^4^ | 4.6x10^4^ | 4x10^4^ | 17.33±6.80 |
| 11 | 3-Br, 4-OCH_3_ | 6x10^4^ | 5.2x10^4^ | 7.4x10^4^ | −24.00±18.18 |
| 12 | 3,5-diBr, 4-OH | 3.2x10^4^ | 4x10^4^ | 5x10^4^ | 18.67±14.73 |
|  | | | | | |
| DMSO control | - | 2 x10^5^ | 2.2x10^5^ | 2.8x10^5^ | 0.00±14.57 |
| 1 | - | 4.4x10^4^ | 1.4x10^5^ | 1.8x10^5^ | 48.00±24.26 |
|  | | | | | |
| DMSO control | - | 1.6x10^5^ | 2x10^4^ | 1x10^5^ | 0.00±66.45 |
| 8 | 3,4-diCl | 0 | 4x10^1^ | 0 | 99.99±0.02 |
| 9 | 3-NO_2_ | 1.8x10^4^ | 2x10^4^ | 3.2x10^4^ | 75.00±6.62 |

**Supplementary Table S2** Primary screening results of ZIKV inhibition

| No. | Substitution (R) | ZIKV titer (pfu/ml) | | Viral Inhibition (%) |
| --- | --- | --- | --- | --- |
|  |  | R1 | R2 |  |
| DMSO control | - | 6x10^4^ | 1x10^5^ | 0.00±25.00 |
| 1 | - | 6x10^4^ | 2x10^4^ | 50.00±25.00 |
|  | | | | |
| DMSO control | - | 1.5x10^5^ | 1.1x10^5^ | 0.00±15.38 |
| 2 | 2-Cl | 6x10^4^ | 2x10^4^ | 69.23±15.38 |
| 3 | 3-Cl | 1600 | 1400 | 98.85±0.08 |
| 4 | 4-Cl | 4x10^3^ | 1.1x10^4^ | 94.23±2.69 |
| 5 | 4-F | 1.9x10^5^ | 1.1x10^5^ | −15.38±30.77 |
| 6 | 4-Br | 3x10^4^ | 4x10^4^ | 73.08 ± 3.85 |
| 7 | 2,4-diCl | 8x10^4^ | 7x10^4^ | 42.31±3.85 |
| 8 | 3,4-diCl | 2x10^2^ | 1x10^2^ | 99.88±0.04 |
| 9 | 3-NO_2_ | 8x10^4^ | 1.6x10^5^ | 7.69±30.77 |
| 10 | 3-Br, 4-OH | 1.6x10^5^ | 1.2x10^5^ | −7.69±15.38 |
| 11 | 3-Br, 4-OCH_3_ | 1.2x10^5^ | 1.4x10^5^ | 0.00±7.69 |
| 12 | 3,5-diBr, 4-OH | 1.4x10^5^ | 1.8x10^5^ | −23.08±15.38 |

**Supplementary Table S3.** Molecular descriptors of Biscoumarin derivatives


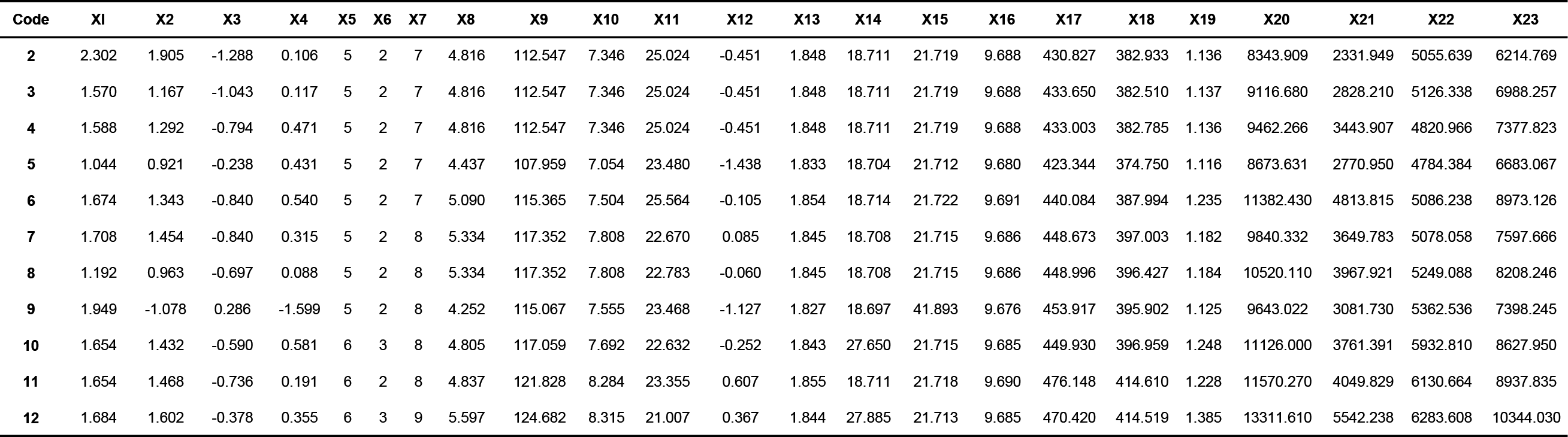


Properties **X1–X23**: Total dipole, Dipole x, Dipole y, Dipole z, Rotatable bonds, Hydrogen bond donor, Hydrogen bond acceptor, AlogP, Molecular refractivity, Molecular flexibility, E-state keys (sums): S_aaCH, E-state keys (sums): S_aasC, E-state keys (sums): S_aaaC, E-state keys (sums): S_sOH, E-state keys (sums): S_dO, E-state keys (sums): S_aaO, Molecular area, Molecular volume, Molecular density, Principal moments of inertia (magnitude), Principal moment of inertia X, Principal moment of inertia Y, and Principal moment of inertia Z (molecular properties calculated using the QSAR model module in the Material Studio program).

**Supplementary Table** S4. The IG values of descriptors in ZIKV.

| **Descriptor** | **GI value** |
| --- | --- |
| Dipole y | 0.264 |
| Molecular area | 0.150 |
| Principal moment of inertia Y | 0.087 |
| E-state keys (sums): S_dO | 0.073 |
| E-state keys (sums): S_aaO | 0.068 |
| E-state keys (sums): S_aaaC | 0.059 |
| Dipole z | 0.046 |
| Rotatable bonds | 0.044 |
| E-state keys (sums): S_aaCH | 0.038 |
| Molecular volume | 0.037 |
| E-state keys (sums): S_sOH | 0.021 |
| Total dipole | 0.015 |
| Principal moment of inertia X | 0.015 |
| Dipole x | 0.014 |
| Molecular density | 0.014 |
| Molecular refractivity | 0.010 |
| Hydrogen bond acceptor | 0.010 |
| AlogP | 0.009 |
| E-state keys (sums): S_aasC | 0.009 |
| Principal moment of inertia Z | 0.003 |
| Principal moments of inertia | 0.001 |
| Molecular flexibility | 0.001 |
| Hydrogen bond donor | 0.001 |

**Supplementary Table** S5. The IG values of descriptors in DENV2.

| **Descriptor** | **GI value** |
| --- | --- |
| Molecular area | 0.122 |
| Dipole y | 0.100 |
| E-state keys (sums): S_aaCH | 0.082 |
| Rotatable bonds | 0.064 |
| Molecular volume | 0.061 |
| Principal moments of inertia | 0.060 |
| E-state keys (sums): S_dO | 0.059 |
| Principal moment of inertia Y | 0.056 |
| E-state keys (sums): S_aasC | 0.054 |
| Total dipole | 0.049 |
| Dipole z | 0.047 |
| E-state keys (sums): S_sOH | 0.039 |
| Molecular refractivity | 0.034 |
| Hydrogen bond donor | 0.031 |
| Molecular density | 0.030 |
| E-state keys (sums): S_aaaC | 0.026 |
| Molecular flexibility | 0.020 |
| AlogP | 0.016 |
| E-state keys (sums): S_aaO | 0.012 |
| Hydrogen bond acceptor | 0.009 |
| Dipole x | 0.008 |
| Principal moment of inertia X | 0.006 |
| Principal moment of inertia Z | 0.003 |

**Supplementary Table S6.** Model performance of Biscoumarin derivatives against DENV2 and ZIKV determined by the RF models

| **Model** | **RF_DENV2_** | **RF_ZIKV_** |
| --- | --- | --- |
| **R^2^** | 0.851 | 0.895 |
| **R^2^_CV_** | 0.749 | 0.829 |
| **RMSE** | 0.290 | 0.288 |

**Supplementary Table S7.** Experimental and predicted log[%inhibition] values of Biscoumarin derivatives against DENV2 and ZIKV

|  |  |  |  |  |
| --- | --- | --- | --- | --- |
| **Code** | **Exp-DENV2** | **Pred-DENV2** | **Exp-ZIKV** | **Pred-ZIKV** |
| **2** | 1.978 | 1.978 | 1.840 | 1.846 |
| **3** | 1.998 | 1.996 | 1.995 | 1.946 |
| **4** | 1.997 | 1.996 | 1.974 | 1.947 |
| **5** | 0.000 | 0.224 | 0.000 | 0.220 |
| **6** | 1.989 | 1.989 | 1.864 | 1.866 |
| **7** | 1.986 | 1.988 | 1.626 | 1.673 |
| **8** | 2.000 | 1.948 | 1.999 | 1.825 |
| **9** | 1.875 | 1.765 | 0.886 | 0.845 |
| **10** | 1.239 | 1.359 | 0.000 | 0.191 |
| **11** | 0.000 | 0.214 | 0.000 | 0.265 |
| **12** | 1.271 | 1.188 | 0.000 | 0.027 |
|  |  |  |  |  |
|  |  |  |  |  |
|  |  |  |  |  |

**Supplementary Table S8.** Δ*G*_bind_ of compound 3 in complex with DENV2 RdRp calculated by MM-PBSA method.

|  | Run 1 | Run 2 | Run 3 |
| --- | --- | --- | --- |
| **Δ*E_electrostatic_*** | -45.83±0.39 | -41.34±0.32 | -38.13±0.48 |
| **Δ*E_vdW_*** | -22.96±0.82 | -22.75±0.77 | -6.62±0.27 |
| **Δ*E_gas_*** | -68.78±0.92 | -64.09±0.81 | -44.75±0.56 |
| **Δ*G_polar_*** | 49.16±0.97 | 44.97±0.66 | 24.82±0.47 |
| **Δ*G_nonpolar_*** | -6.56±0.05 | -6.05±0.02 | -5.88±0.03 |
| **Δ*G_bind (PBSA)_*** | 42.60±0.94 | 38.92±0.66 | 18.94±0.47 |
| **Δ*G_bind_ (MM/PBSA)*** | -26.18±0.41 | -25.17±0.32 | -25.81±0.49 |

**Supplementary Table S9.** Δ*G*_bind_ of compound 4 in complex with DENV2 RdRp calculated by MM-PBSA method.

|  | Run 1 | Run 2 | Run 3 |
| --- | --- | --- | --- |
| **Δ*E_electrostatic_*** | -39.62±0.45 | -40.71±0.21 | -36.90±0.25 |
| **Δ*E_vdW_*** | -16.86±0.64 | -21.43±0.38 | -6.47±0.49 |
| **Δ*E_gas_*** | -56.48±0.75 | -62.14±0.44 | -43.36±0.55 |
| **Δ*G_polar_*** | 38.48±0.86 | 42.22±0.50 | 32.75±0.69 |
| **Δ*G_nonpolar_*** | -5.88±0.03 | -5.60±0.03 | -6.57±0.02 |
| **Δ*G_bind (PBSA)_*** | 32.60±0.85 | 36.62±0.49 | 26.18±0.68 |
| **Δ*G_bind_ (MM/PBSA)*** | -23.88±0.59 | -25.51±0.35 | -17.18±0.42 |


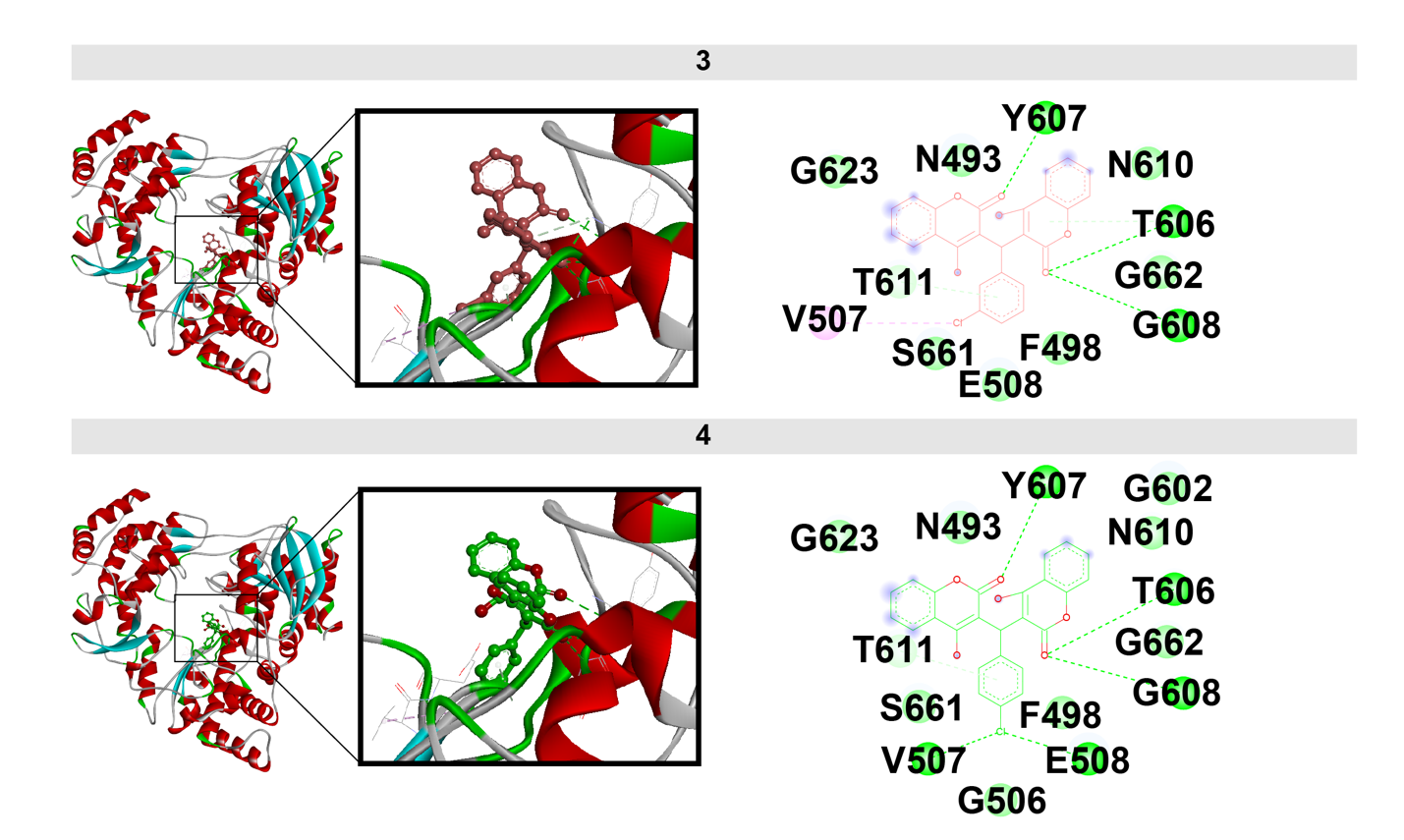


**Supplementary Fig. S1** Molecular docking result of compounds 3 and 4 binding to the RNA tunnel of DENV2 RdRp.


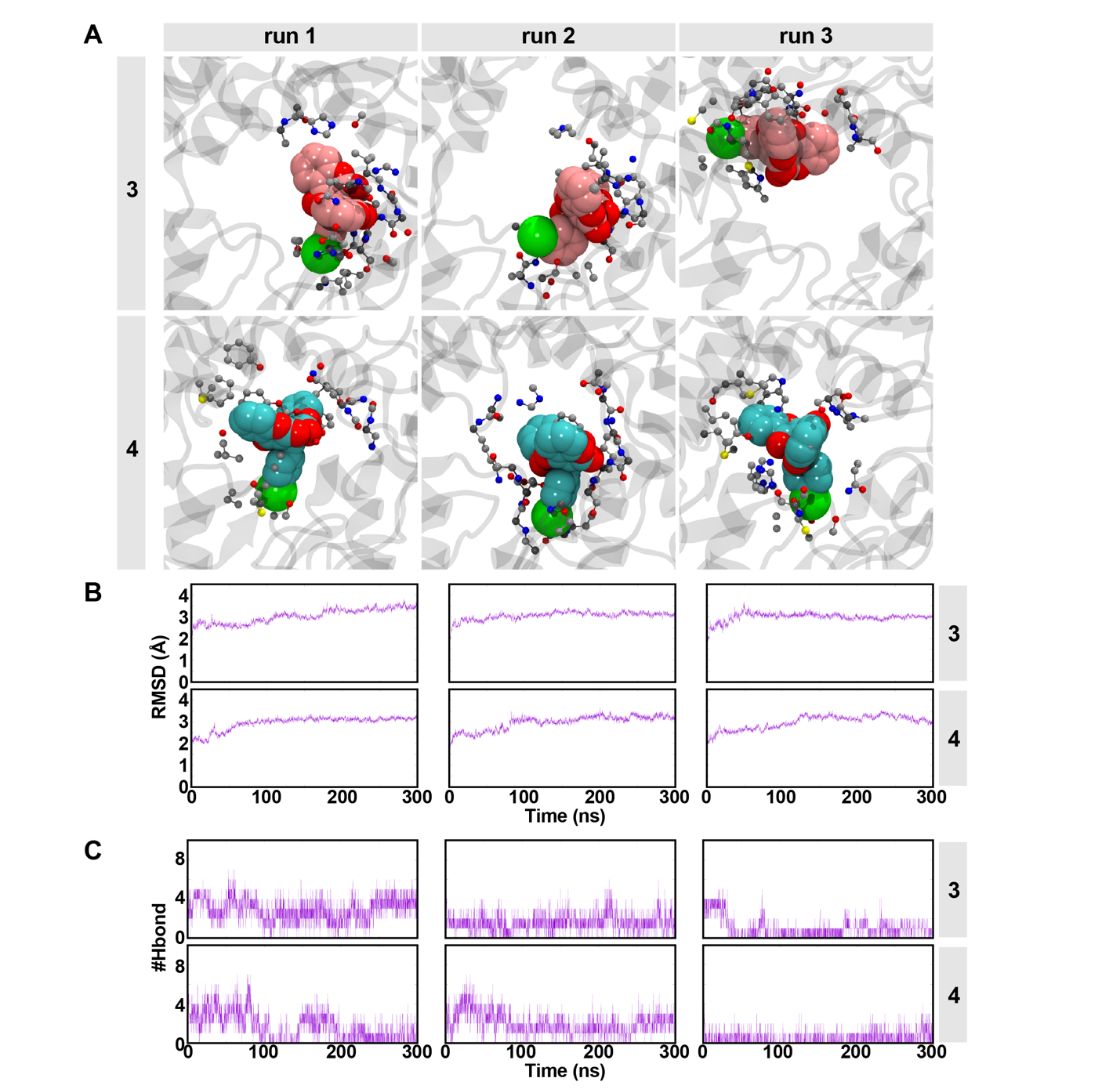


**Supplementary Fig. S2** Binding dynamics of compounds 3 and 4 within the RNA tunnel of DENV2 RdRp over 300 ns MD simulations. (A) Representative snapshots from three independent MD replicates (run 1–3) show the binding poses of compound 3 (top row, red spheres) and compound 4 (bottom row, cyan spheres) within the RNA tunnel (grey ribbon). Key residues are shown as sticks, and Cl atoms from the compounds are depicted as green spheres. (B) Time evolution of ligand RMSD (Å) for each replicate of compounds 3 (top) and 4 (bottom), indicating the structural stability of the bound ligands. (C) Number of hydrogen bonds between each compound and the protein over time, illustrating the persistence of protein-ligand interactions. Compound 3 shows more consistent stability and hydrogen bonding compared to compound 4 across replicates.


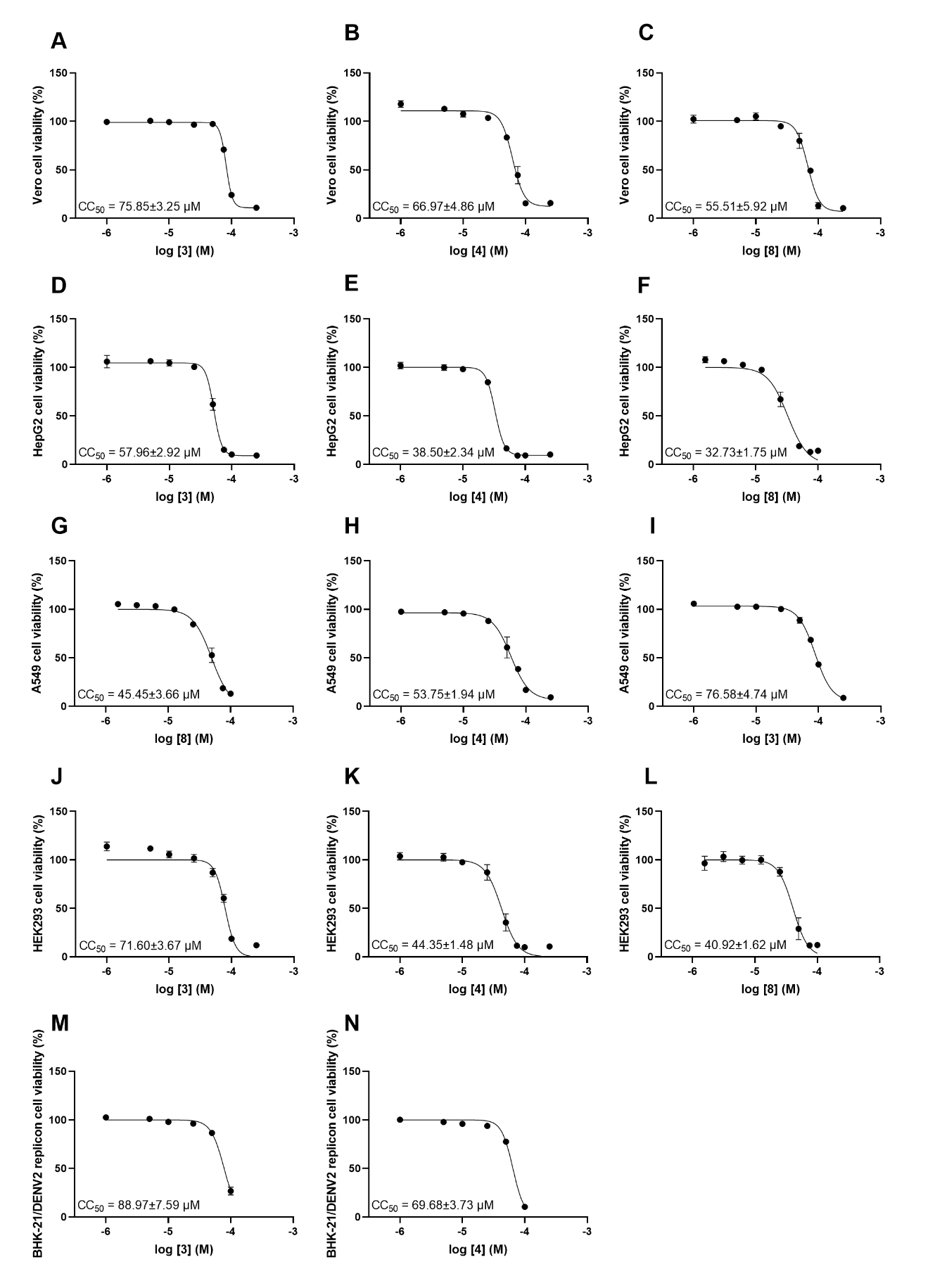


**Supplementary Fig. S3** CC_50_ of compounds 3, 4, and 8 in various cell lines.


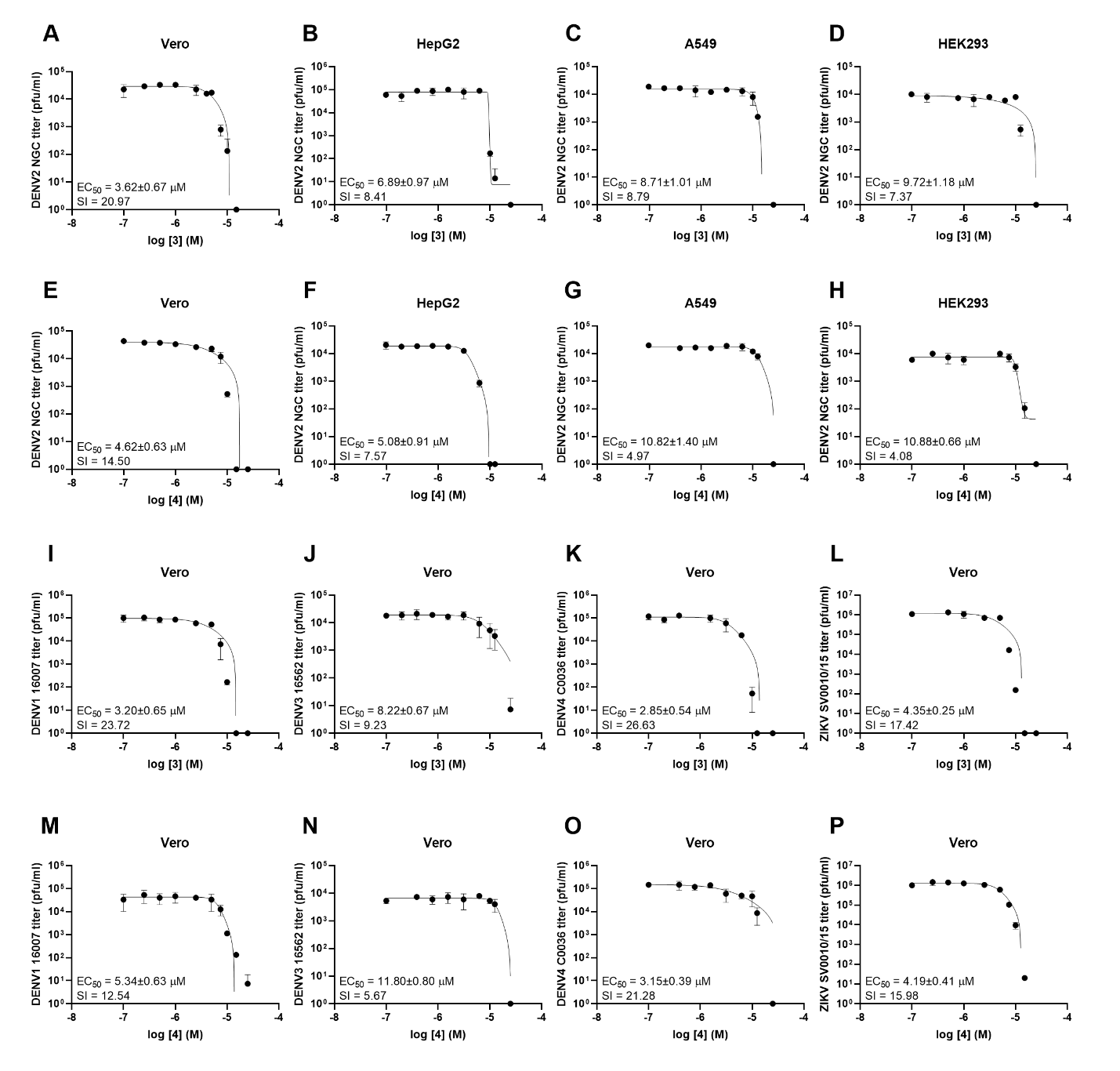


**Supplementary Fig. S4** EC_50_ of compounds 3, and 4 against DENV1-4 and ZIKV in various cell lines.

**
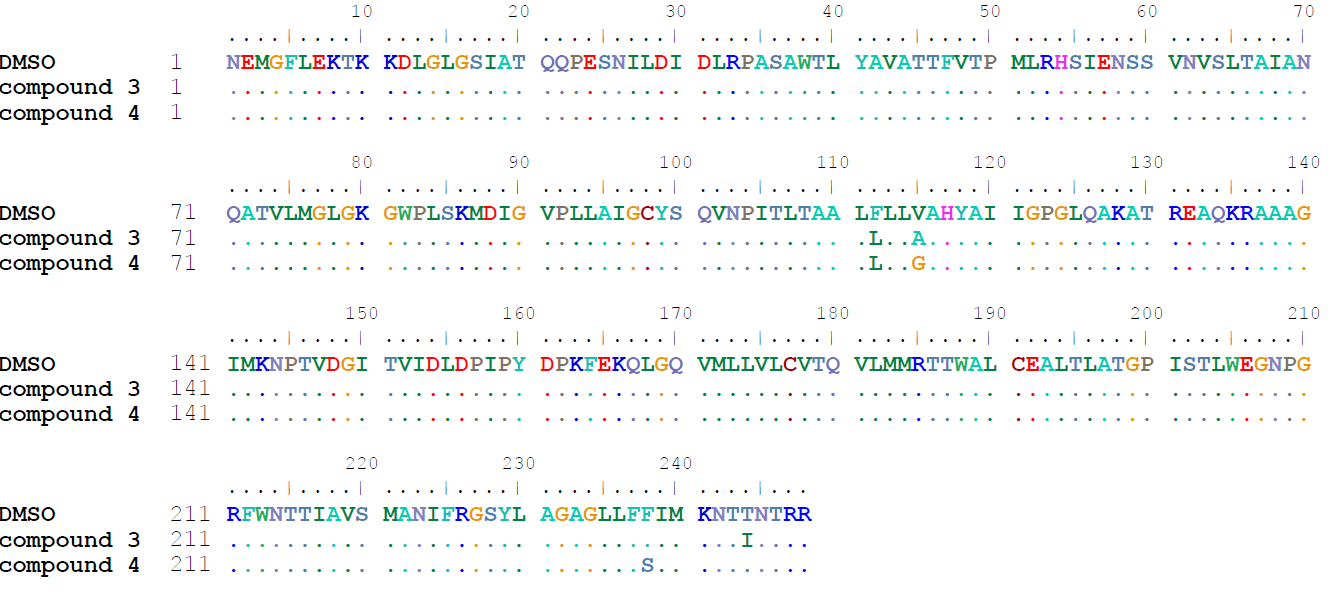
**

**Supplementary Fig. S5 Amino acid sequences in NS4B of mutant DENV2**


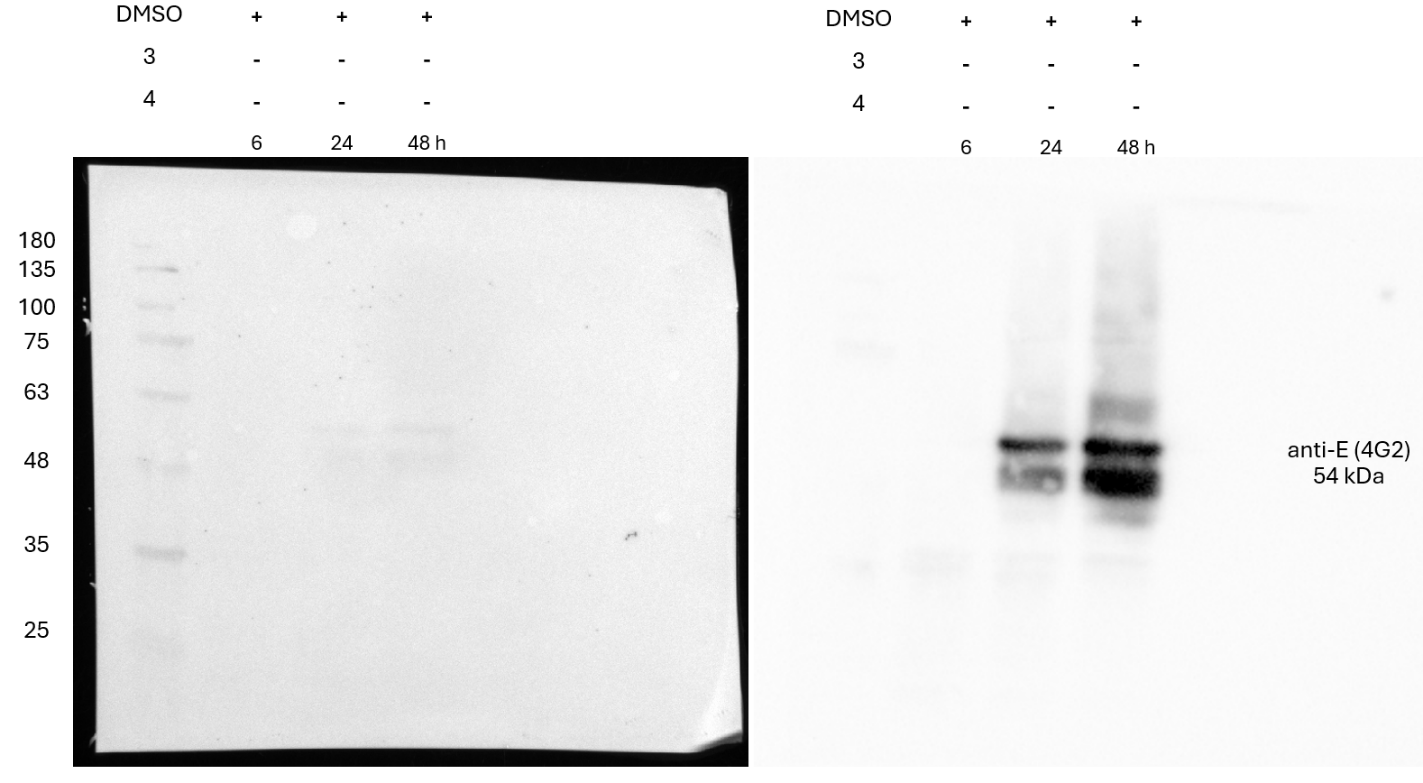

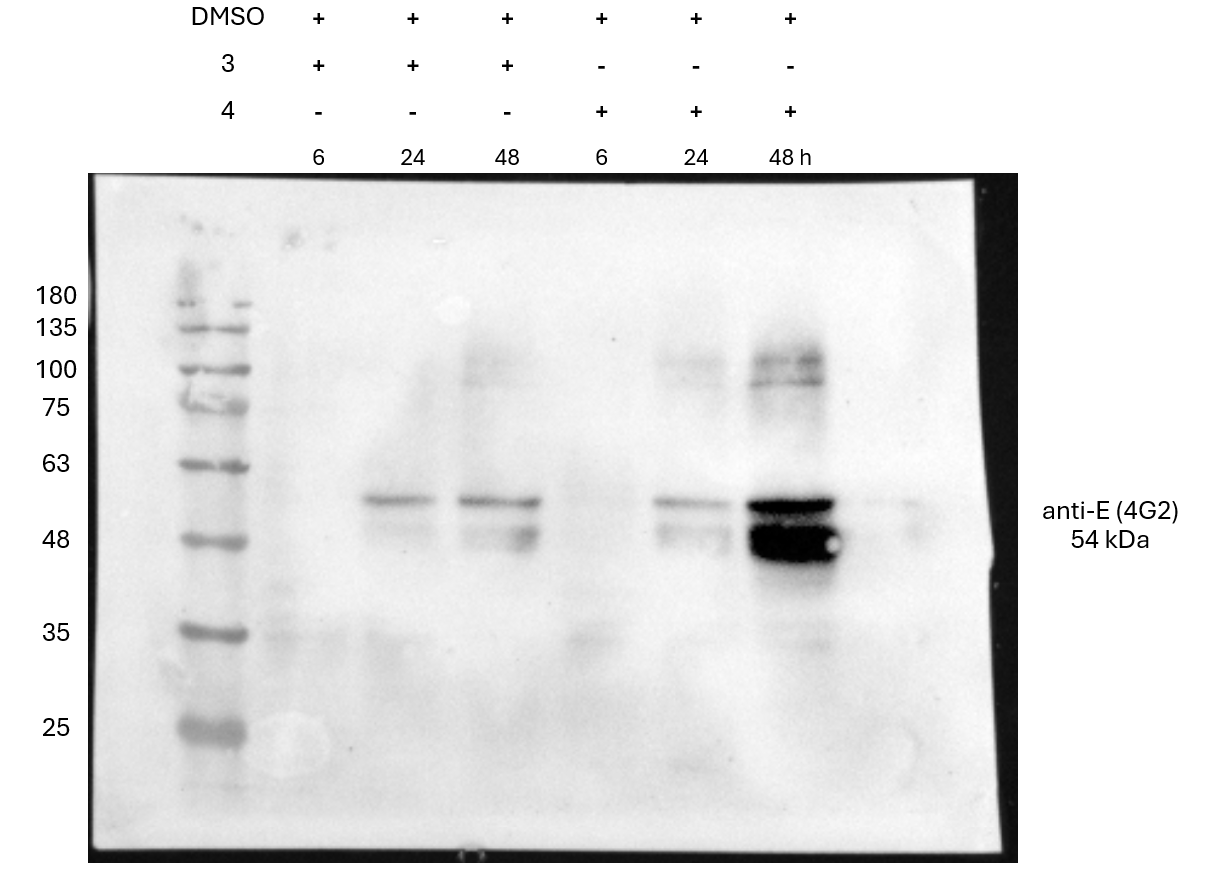

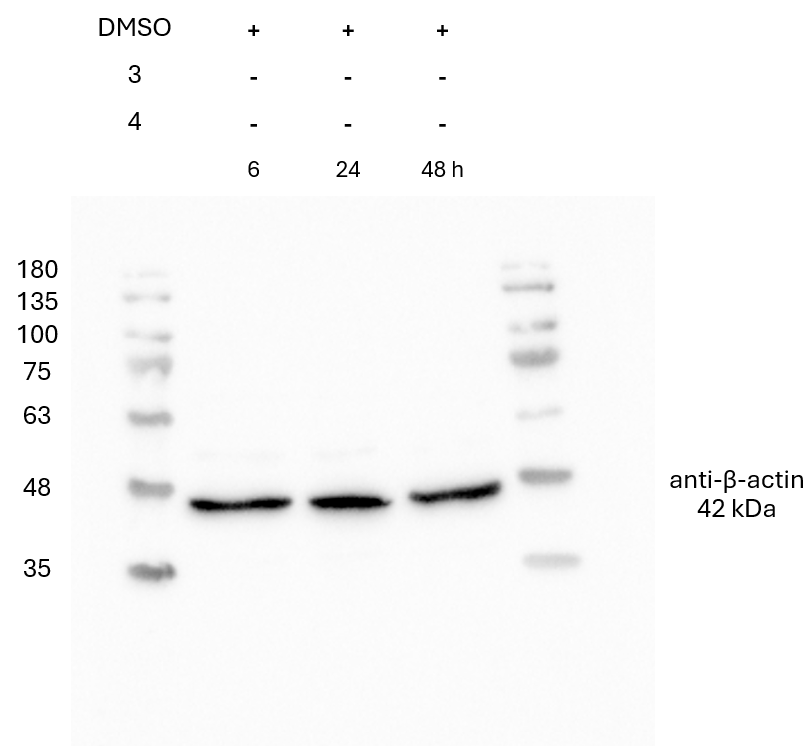

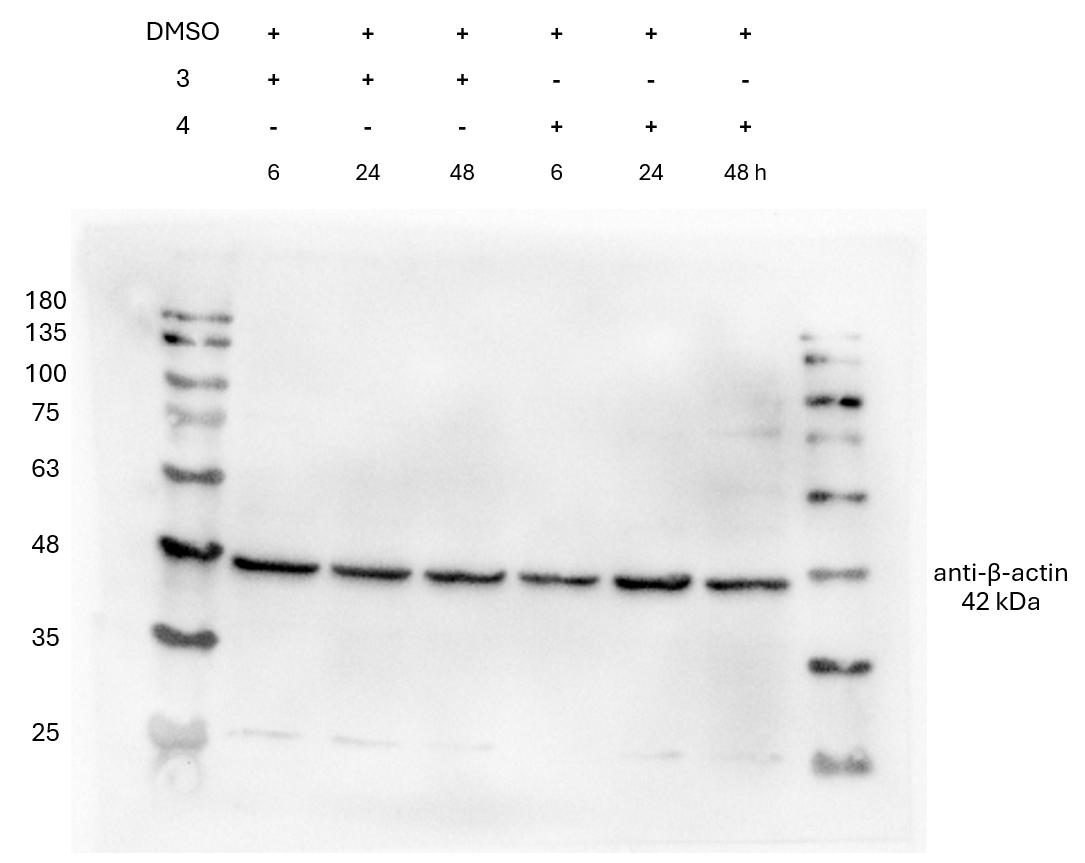


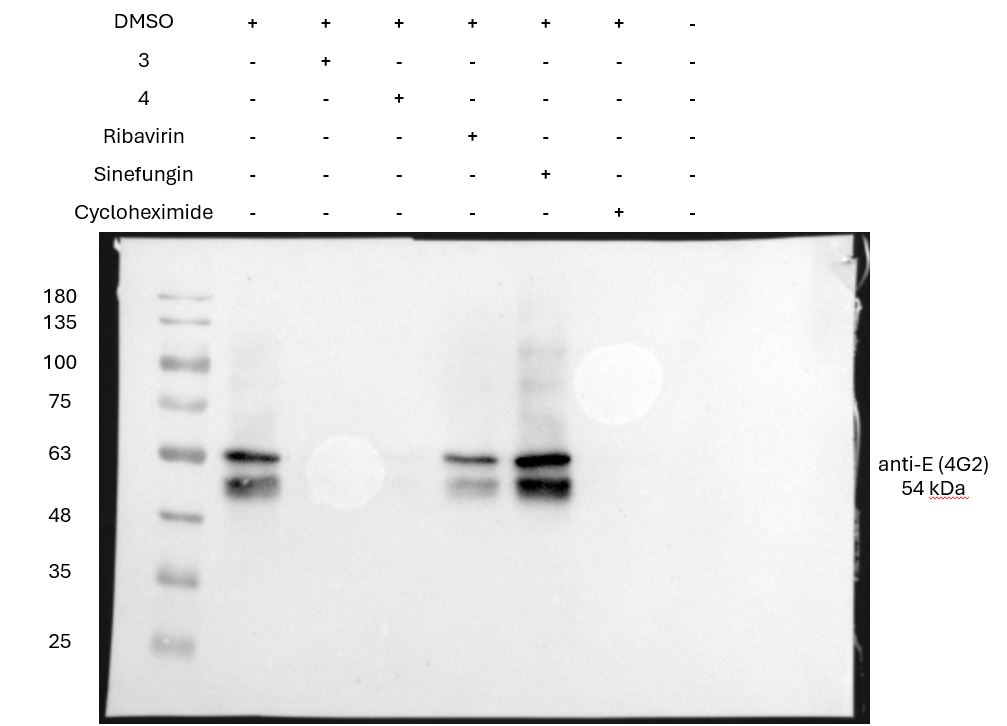

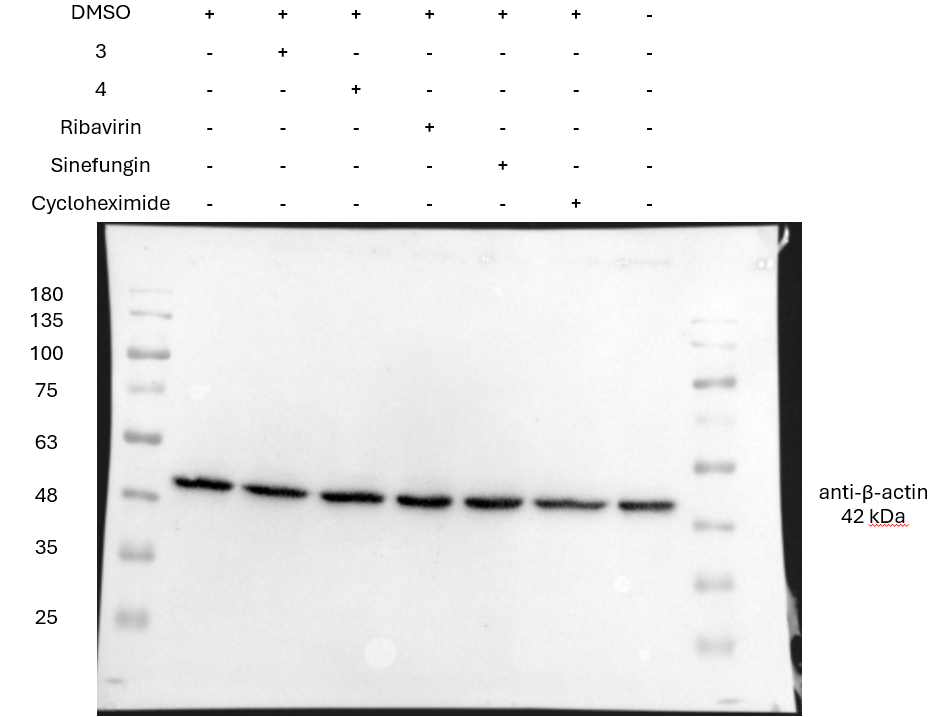


**Supplementary Fig. S6** Full WB blot picture
